# Organelle-tuning condition robustly fabricates energetic mitochondria for cartilage regeneration

**DOI:** 10.1038/s41413-025-00411-6

**Published:** 2025-03-17

**Authors:** Xuri Chen, Yunting Zhou, Wenyu Yao, Chenlu Gao, Zhuomin Sha, Junzhi Yi, Jiasheng Wang, Xindi Liu, Chenjie Dai, Yi Zhang, Zhonglin Wu, Xudong Yao, Jing Zhou, Hua Liu, Yishan Chen, Hongwei Ouyang

**Affiliations:** 1 Department of Sports Medicine of the Second Affiliated Hospital, and Liangzhu Laboratory, Zhejiang University School of Medicine, Hangzhou, China; 2 Dr. Li Dak Sum & Yip Yio Chin Center for Stem Cells and Regenerative Medicine, Zhejiang University School of Medicine, Hangzhou, China; 3 Center of Regenerative and Aging Medicine, the Fourth Affiliated Hospital of School of Medicine, and International School of Medicine, International Institutes of Medicine, Zhejiang University, Yiwu, China; 4 Zhejiang University-University of Edinburgh Institute, Zhejiang University School of Medicine, Haining, China; 5 China Orthopedic Regenerative Medicine Group (CORMed), Hangzhou, China

**Keywords:** Bone, Energy metabolism

## Abstract

Mitochondria are vital organelles whose impairment leads to numerous metabolic disorders. Mitochondrial transplantation serves as a promising clinical therapy. However, its widespread application is hindered by the limited availability of healthy mitochondria, with the dose required reaching up to 10^9^ mitochondria per injection/patient. This necessitates sustainable and tractable approaches for producing high-quality human mitochondria. In this study, we demonstrated a highly efficient mitochondria-producing strategy by manipulating mitobiogenesis and tuning organelle balance in human mesenchymal stem cells (MSCs). Utilizing an optimized culture medium (mito-condition) developed from our established formula, we achieved an 854-fold increase in mitochondria production compared to normal MSC culture within 15 days. These mitochondria were not only significantly expanded but also exhibited superior function both before and after isolation, with ATP production levels reaching 5.71 times that of normal mitochondria. Mechanistically, we revealed activation of the AMPK pathway and the establishment of a novel cellular state ideal for mitochondrial fabrication, characterized by enhanced proliferation and mitobiogenesis while suppressing other energy-consuming activities. Furthermore, the in vivo function of these mitochondria was validated in the mitotherapy in a mouse osteoarthritis model, resulting in significant cartilage regeneration over a 12-week period. Overall, this study presented a new strategy for the off-the-shelf fabrication of human mitochondria and provided insights into the molecular mechanisms governing organelle synthesis.

## Introduction

Cells contain various sophisticated organelles that act as intracellular biofactories by transforming chemical energy and substances. Among these, the mitochondria are particularly indispensable, providing 90% of the bioenergy required for human life. Thus, their dysfunction inevitably leads to pathological conditions.^1–5^ When cells fail to compensate for mitochondrial damages, organelle transplantation becomes an alternative approach to restore tissue homeostasis, serving as more precise and less immunogenic management compared to cell transplantation.^6^ The advantages of organelle transplantation have been implied in several studies.^7,8^ For example, one study employed plant-derived nanothylakoids to precisely modulate the metabolic disorder in osteoarthritis (OA),^9^ a prevalent degenerative joint disease.^10^

Given the crucial role of mitochondria in metabolic wellness, artificial mitochondrial transplantation has been developed for managing a wide range of diseases. For instance, mitochondrial transplantation has been shown to be an effective therapy for improving cardiac function and alleviating symptoms of ischaemic heart disease,^11–13^ a leading cause of disability and death^14^ characterized by mitochondrial dysfunction in cardiomyocytes. This strategy has also been advantageous in the treatment of mitochondrial DNA (mtDNA) disorders,^15^ infertility,^16^ and wound healing,^17^ where healthy mitochondria from donor cells are injected into the disease tissues to replace or fuse with the dysfunctional mitochondria. Additionally, given the increasing global burden of metabolic diseases, this approach has been proposed for widespread application in metabolic syndrome as a promising strategy for maintaining metabolic homeostasis and restoring energy supply.^18–20^ However, despite the broad potential application, a significant challenge remains in harvesting safe and healthy mitochondria resources. Due to their complex double-membrane structure and intricate chemical machinery, de novo artificial synthesis of mitochondria is exceedingly difficult.^21,22^ Consequently, mitochondria-rich human tissues, such as the liver and muscle, currently serve as the major resource for donor mitochondria in clinical use. Nevertheless, the mitochondria invasively isolated from non-expandable tissues^23^ cannot sustainably meet the growing demand for mitotherapy (e.g., over 10^9^ mitochondria for one ischemia-reperfusion injury patient).^24^ Additionally, the quality of mitochondria obtained in this manner cannot be adequately controlled, as it varies with donors’ age^25^ and health status.^15^ Consequently, a significant gap exists between the current mitochondrial harvesting strategy and the need for highly efficient mitochondrial fabrication to meet clinical demands. Therefore, it is imperative to develop new strategies that guarantee sustainable and controllable production of high-quality human mitochondria.

To address this issue, stem cells provide a good platform for off-the-shelf cell derivative fabrication due to their self-renewal capability in long-term culture,^26–28^ while cell engineering enables the manipulation of stem cell fate and function.^29–32^ For example, in our previous studies, we have successfully developed a customized serum-free culture system to rapidly expand human adipose-derived mesenchymal stem cells (MSCs) to provide cellular resources in tissue regeneration.^29^ However, biofabrication of mitochondria presents greater challenges, requiring sophisticated organelle-level manipulations. This is especially true given our limited understanding and the fact that mitochondria, unlike secreted exosomes, are essential intracellular components crucial for cell survival. Furthermore, mitochondrial synthesis involves the coordinated cooperation of numerous other organelles (such as the nucleus, ribosomes, and transport vesicles) within a complex regulatory network. As a result, it is challenging yet essential to prevent an imbalance in bioenergy flow or overloading of a single organelle type during live-cell mitochondrial fabrication. This may be addressed by establishing a new metabolic balance or tuning the overall cellular regulation of organelle synthesis. Moreover, to achieve a producible and sustainable mitochondrial fabrication, cell expansion must be facilitated in an optimal culture that simultaneously improves both cell proliferation and mitobiogenesis.

In this study, we proposed a novel strategy for efficient large-scale fabrication of human mitochondria by identifying a customized mitochondria-producing condition (mito-condition). We used human adipose-derived MSCs, which were effective cell sources for OA therapy, can be conveniently obtained from human donors, and can be stably expanded in long-term culture.^33^ Through phenotypic screening and optimization based on our previous study, we developed a mito-condition that significantly enhanced both mitochondrial quantity and quality in MSC culture. The sustainable production was achieved by continuous cell passage, resulting in a final mitochondria yield ~ 854-fold greater than the initial cells after 5 passages. This approach enables the production of ~10^13^ off-the-shelf mitochondria within 15 days. In addition to the increased quantity, the optimized mitochondria demonstrated robust bioenergetic function in mitochondrial assays, exhibiting a 5.71-fold readout compared to normal MSCs. Using transcriptomics and other validations, we uncovered that the mito-condition regulated cell mitobiogenesis by activating the Adenosine 5‘-monophosphate (AMP)-activated protein kinase (AMPK) pathway and modifying the balance between energy-generating and energy-consuming programs. In vivo, our tailor-made mitochondria were applied in a mouse OA model, successfully promoting cartilage regeneration and ameliorating OA. Overall, this study provides a new perspective on the artificial manipulation of cellular organelle synthesis and lays a foundation for the development of mitochondrial engineering techniques in regenerative medicine.

## Results

### A combinatorial phenotypic screen identifies mito-condition for robust mitochondria production

**Fig. 1:**
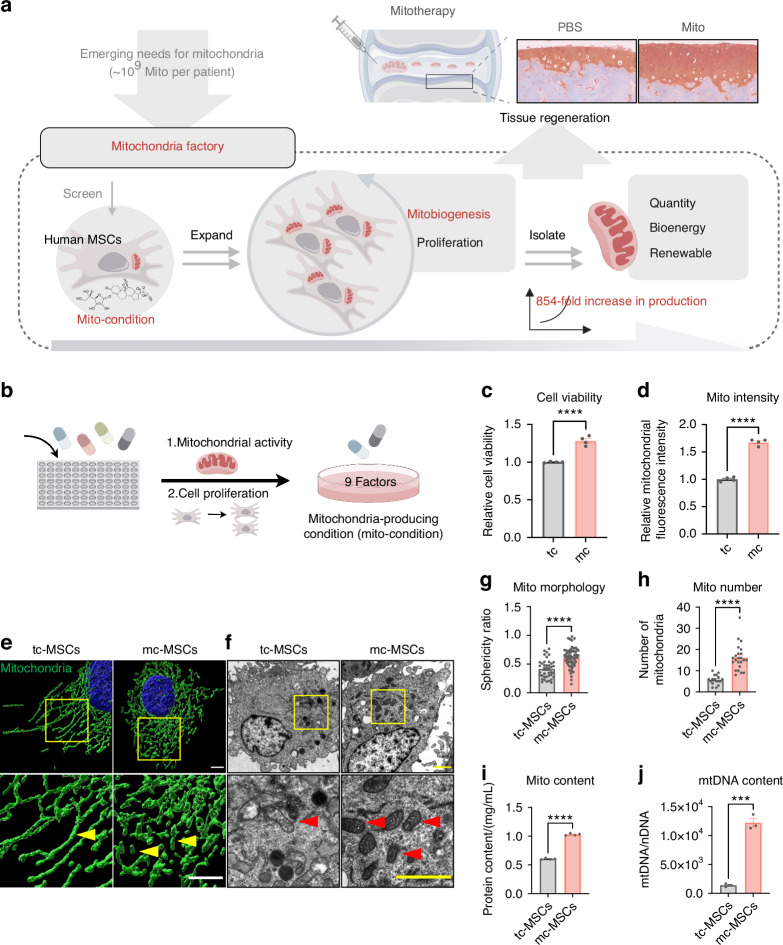
A combinatorial phenotypic screen identifies mito-condition for robust mitochondria production. **a** Schematic illustration of mitochondrial production for cartilage regeneration. **b** Schematic illustration of the screening of the ingredients of the medium. **c** The relative cell viability of tc-MSCs and mc-MSCs (well = 4). **d** The relative mitochondrial intensity of tc-MSCs and mc-MSCs (well = 4). **e** Representative images of mitochondria in tc-MSCs and mc-MSCs. Scale bar, 5 μm. Yellow arrows: mitochondria. **f** Representative transmission electron microscope (TEM) images of tc-MSCs and mc-MSCs. Scale bar, 2 μm. Red arrows: mitochondria. **g** Sphericity ratio of mitochondria of tc-MSCs and mc-MSCs from TEM images (54 and 40 mitochondria were calculated for tc-MSCs and mc-MSCs group respectively). **h** Number of mitochondria tc-MSCs and mc-MSCs from TEM images (17 and 23 cells were calculated for tc-MSCs and mc-MSCs group respectively). **i** Quantification of mitochondrial protein content (well = 3). **j** Quantification of the copy number of mtDNA (well = 3). All data are presented as mean ± SEM. ***P < 0.001, ****P < 0.000 1. *P* values were determined using unpaired two-tailed t-test (**c**, **d**, **g**–**j**)

To construct a mitochondrial fabrication platform within MSCs, we performed a phenotypic screen focusing on cell proliferation and mitochondrial activity (Fig. 1a). Building upon our previously established serum-free culture expansion system which yielded MSCs with high proliferative capacity, maintained stemness and low immunogenicity comparable to serum cultured MSCs,^29^ we conducted a combinatorial screening to optimize the culture medium. By systematically excluding components that did not contribute to mitochondrial biogenesis (mitobiogenesis), we identified 8 factors that positively influenced cell proliferation and total mitochondrial content (Fig. 1b and Fig. S1a). To further enhance the mitochondrial content, we incorporated human platelet lysate (HPL), a commonly used serum alternative known to promote both cell proliferation and mitochondrial function.^34–36^ The addition of HPL to the 8 factors medium significantly increased the total mitochondrial content in MSCs (Fig. S1b). Consequently, our optimized mito-condition medium comprised nine factors: basic fibroblast growth factor (bFGF), sodium bicarbonate (NaHCO_3_), lipid concentrate, Insulin-Transferrin-Selenium (ITS), progesterone (Prog), hydrocortisone (Hc), Vitamin C (Vc), heparin sodium (HS) and HPL. Notably, 4 factors of these factors, bFGF, Hc, Vc, and HPL, have been previously reported to promote mitochondrial biogenesis.^37–39^ We also validated the contribution of each component by individually removing them from the medium; omission of any single factor led to a significant decrease in mitochondrial intensity (Fig. S1c). With this optimized culture medium, we established conditions conducive to mitochondrial fabrication and designated the cultured MSCs as mito-condition MSCs (mc-MSCs). These mc-MSCs exhibited significantly higher cell proliferation rates and mitochondrial quantities compared to MSCs cultured under typical serum-containing conditions (tc-MSCs) (Fig. 1c, d).

To validate the mitochondrial phenotype in mc-MSCs, we characterized their mitochondrial morphology and content. High-resolution images of the mitochondrial outer membrane protein TOMM20 revealed that mitochondria in mc-MSCs predominantly exhibited a short and rounded morphology, in contrast to the elongated mitochondria in tc-MSCs (Fig. 1e). These morphology features were further confirmed by the transmission electron microscopy (TEM) analyses (Fig. 1f, g). Quantitive analysis showed that mc-MSCs contained significantly increased amounts of mitochondria (2.83-fold), mitochondrial proteins (1.70-fold), and mtDNAs copies (8.69-fold) per cell on average compared to tc-MSCs (Fig. 1h–j).

Overall, these data indicated that our screening successfully identified a mito-condition that enhanced both cell proliferation and total mitochondrial quantity in MSC culture.

### The mito-condition maintains sustainable mitochondrial reproduction during stem cell passaging

**Fig. 2:**
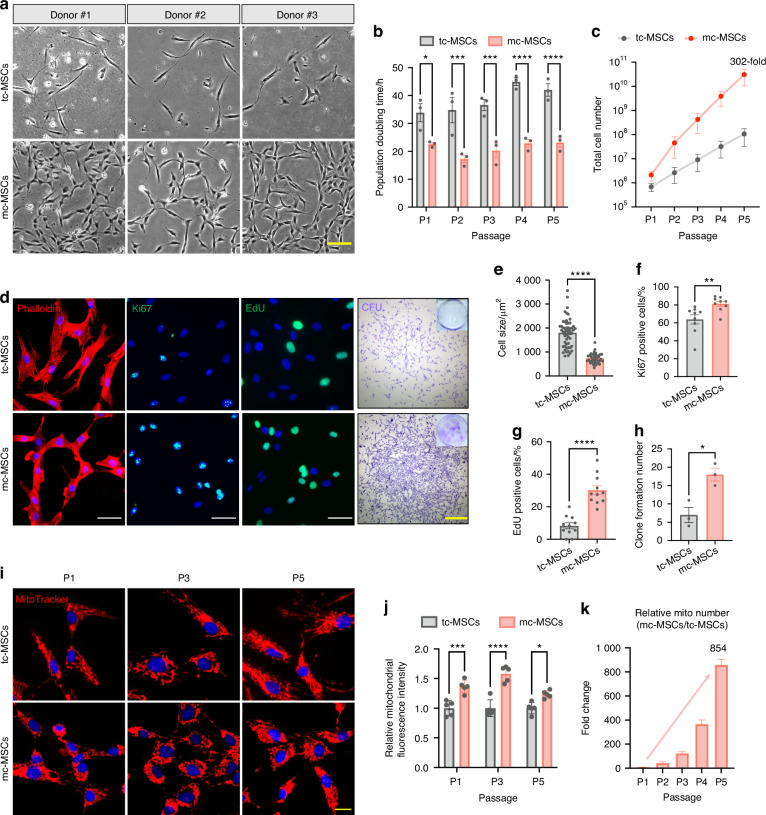
The mito-condition maintains sustainable mitochondrial reproduction during stem cell passaging. **a** Representative bright field images of primary cells from 3 donors. Scale bar, 20 μm. **b** PDT of tc-MSCs and mc-MSCs at P1-P5 (sample = 3 from different donors). **c** Total cell number of tc-MSCs and mc-MSCs at P1-P5 (sample = 3 from different donors). **d** Representative images of phalloidin staining, immunostaining of Ki67 and EdU, and CFU. Scale bar, 100 μm (for Ki67 and EdU) and 1 mm (for CFU). **e** Relative cell size of tc-MSCs and mc-MSCs (well = 3 for each group, 54 and 40 cells were calculated for tc-MSCs and mc-MSCs group respectively). **f** Percent of Ki67 positive cells in tc-MSCs and mc-MSCs (cells in 9 fields of view from 3 wells were calculated). **g** Percent of EdU positive cells in tc-MSCs and mc-MSCs (cells in 11 fields of view from 3 wells were calculated). **h** The number of colonies of tc-MSCs and mc-MSCs (well = 3). **i** Representative images of Mito-Tracker labeled mitochondria in tc-MSCs and mc-MSCs at P1, P3, and P5. Scale bar, 20 μm. **j** Relative total MitoTracker fluorescence intensity in tc-MSCs and mc-MSCs at P1, P3, and P5 (well = 5). Cells at different passages were seeded with the same initial density and compared after a 3-day culture. **k** Relative mitochondrial number change of mc-MSCs/tc-MSCs at different passages (sample = 3 from different donors). All data are presented as mean ± SEM. *P < 0.05, **P < 0.01, ***P < 0.001, ****P < 0.000 1. *P* values were determined using unpaired two-tailed *t*-test (**b**, **c**, **e**-**h**, **j**)

Building upon our findings that the mito-condition enhanced both cell proliferation and mitobiogenesis, we explored whether sustainable mitochondrial reproduction could be achieved during MSC passaging. Data from MSCs derived from the different donors (*n* = 3) demonstrated that cells cultured under the mito-condition proliferated significantly faster than those in typical conditions (Fig. 2a). The population doubling time (PDT) of mc-MSCs was 21.23 h, markedly shorter than the 38.45 h observed for tc-MSCs (Fig. 2b). After 5 passages (~15 days) of expansion, the total number of mc-MSCs was 302 times greater than that of tc-MSCs (Fig. 2c). Morphological analysis using F-actin cytoskeleton staining revealed that mc-MSCs had a reduced cell size, consistent with their robust proliferative phenotype (Fig. 2d, e). Immunostaining for proliferation markers Ki67 and 5-ethynyl-2′-deoxyuridine (EdU) staining displayed a higher proportion of proliferative cells in mc-MSCs compared to tc-MSCs (Ki67: 81.29% vs. 63.88%, EdU: 30.20% vs. 8.24%) (Fig. 2f, g), corroborated by flow cytometric data (28.35% vs. 7.86%) (Fig. S2a). Besides, the cell cycle assay showed an increased S phase proportion in mc-MSCs (Fig. S2b). In the colony-forming unit assays assessing self-renewal ability, mc-MSCs formed significantly more and larger colonies than tc-MSCs (Fig. 2d, h). While both tc-MSCs and mc-MSCs positively expressed standard MSC surface markers (Fig. S3a), mc-MSCs exhibited higher muti-lineage differentiation potential (Fig. S3b, c).

Comparative analyses across different passages confirmed that mc-MSCs stably consistently maintained a higher mitochondrial quantity than tc-MSCs (Fig. 2i). Specifically, the relative Mito-Tracker intensity in mc-MSCs at passage 1, 3 and 5 were 1.36-fold, 1.58-fold and 1.23-fold higher, respectively, compared to tc-MSCs (Fig. 2j). After 5 passages over 15 days, the estimated total mitochondrial yield from mc-MSCs was at least 854-fold greater than that from tc-MSCs. This was calculated by multiplying the fold increase in mitochondria per cell (from Fig. 1h) by the fold increase in total cell number (from Fig. 2c); thus, 302 × 2.83 = 854 (Fig. 2k). This substantial increase demonstrates the efficacy of the mito-condition as a powerful methodology for large-scale synthesis of human mitochondria.

### The mito-condition improves mitochondrial function for a robust energy supply

**Fig. 3:**
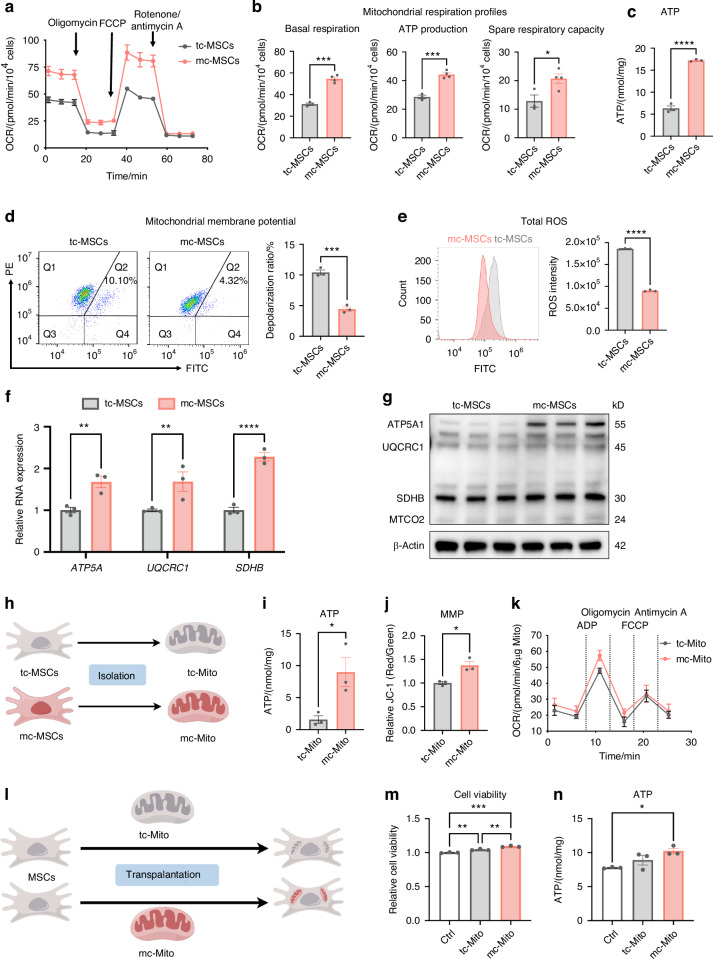
The mito-condition produces energetic mitochondria for a robust energy supply. **a** OCR assay of tc-MSCs and mc-MSCs (replicate = 4). **b** Quantitative analysis of mitochondrial respiration profiles (well = 4). **c** ATP content in tc-MSCs and mc-MSCs (well = 3). **d** JC-1 staining followed by flow cytometric analysis and depolarization ratio of tc-MSCs and mc-MSCs (well = 3). **e** Analysis of ROS intensity through flow cytometry and quantification in tc-MSCs and mc-MSCs (replicate = 3). **f** Gene expression anylysis of OXPHOS-related genes (*ATP5A1*, *UQCRC1*, and *SDHB*) in tc-MSCs and mc-MSCs (well = 3). **g** Western blot (WB) analysis of OXPHOS-related proteins (ATP5A1, UQCRC1, SDHB, and MTCO2) expression in tc-MSCs and mc-MSCs. **h** Schematic diagram of mitochondria isolated from tc-MSCs and mc-MSCs. **i** ATP content of tc-Mito and mc-Mito (well = 3). **j** Quantification of the JC-1 fluorescence ratio of tc-Mito and mc-Mito (well = 3). **k** OCR assay of tc-Mito and mc-Mito (well = 12). **l** Schematic illustration of the transplantation of tc-Mito and mc-Mito into MSCs. **m** Relative cell viability of MSCs after mitochondrial transplantation (well = 3). **n** ATP content of MSCs after (well = 3). All data are presented as mean ± SEM. *P < 0.05, **P < 0.01, ***P < 0.001, ****P < 0.000 1. *P* values were determined using unpaired two-tailed *t*-test (**b**-**f**, **i**, **j**) or one-way ANOVA (**f**, **m**, **n**)

Beyond increasing mitochondrial quantity, we investigated whether the mito-condition boosted mitochondrial metabolic activity. The bioenergetics measurements using the Seahorse extracellular flux analyzer recorded a significant increase in mitochondrial respiration levels in mc-MSCs (Fig. 3a). The data demonstrated enhanced basal respiration, ATP production, and spare respiration capacity (Fig. 3b). Consistently, ATP assay results showed that mc-MSCs produced higher levels of total cellular ATP compared to tc-MSCs (Fig. 3c). In addition, mc-MSCs demonstrated superior performance in assessments of mitochondrial membrane potential (MMP) and reactive oxygen species (ROS). The flow cytometric analysis revealed that mc-MSCs displayed a decreased mitochondrial depolarization ratio (4.32% vs. 10.41%) and reduced ROS accumulation (0.9 × 10^5^ vs. 1.8 × 10^5^) compared to tc-MSCs (Fig. 3d, e). Furthermore, we examined the expression levels of representative functional genes (*ATP5A1*, *UQCRC1*, and *SDHB*) and proteins (ATP5A1, UQCRC1, SDHB, and MTCO2) involved in oxidative phosphorylation (OXPHOS) and demonstrated their levels were increased in mc-MSCs compared to tc-MSCs (Figs. 3f, g and S4). These findings confirmed that mc-MSCs exhibited improved mitochondrial function under the mito-condition culture.

To verify that these energetic mitochondria preserved their function during mitochondrial transplantation procedures, we compared the activity of isolated mitochondria from mc-MSCs and tc-MSCs (Fig. 3h). Post-isolated, mc-mitochondria produced elevated levels of ATP and displayed higher MMP (Fig. 3i, j). The analysis of ADP-driven mitochondrial activity demonstrated a slight increase in the measured OCR of mc-Mito compared to tc-Mito (Fig. 3k). Notably, these energetic mitochondria remained enhanced MMP after 24 h of storage at 4°C or 37°C (Fig. S5a), indicating their advantageous capacity to maintain function in vitro. Besides, we detected the ATP production levels of mc-mitochondria at different time points and found that the function of mc-mitochondria stored at 4°C was more stable than those stored at 37°C (Fig. S5b), indicating a preferred storage temperature for clinical practices. Furthermore, after transplanting these mitochondria into other MSCs, we observed that mc-mitochondria could boost both cell proliferation and ATP production in the recipient cells (Fig. 3l–n).

In summary, the mito-condition improved the metabolic function of mitochondria in mc-MSCs. These mitochondria produced higher levels of ATP before and after isolation, potentially ensuring improved outcomes in clinical mitochondrial transplantation.

### The mito-condition improves mitobiogenesis by activating the AMPK pathway

**Fig. 4:**
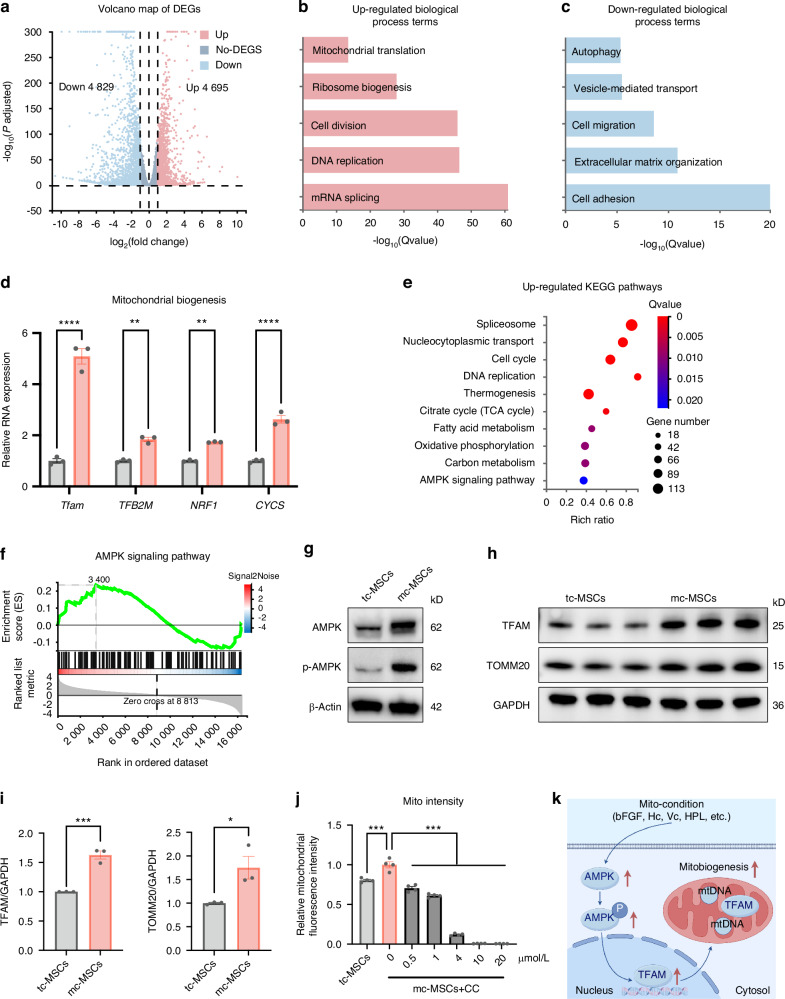
The mito-condition creates a sustainable energy-generating homeostasis by activating the AMPK pathway. **a** Volcano plots showing differently expressed genes between tc-MSCs and mc-MSCs. **b** Up-regulated GO terms. **c** Down-regulated GO terms. **d** Relative mRNA expression of mitobiogenesis-related genes from RNA-seq data. **e** Upregulated KEGG pathways. **f** The GSEA analysis of the AMPK signaling pathway. **g** Western blot (WB) analysis of AMPK and p-AMPK expression in tc-MSCs and mc-MSCs. **h** WB analysis of TFAM and TOMM20 expression in tc-MSCs and mc-MSCs. **i** Quantification of WB results in (**h**) (well = 3). **j** The relative mitochondrial intensity of MSCs after being treated with CC (well = 3). **k** Schematic diagram showing the mito-condition promotes mitochondrial biogenesis by the AMPK pathway. All data are presented as mean ± SEM. *P < 0.05, **P < 0.01, ***P < 0.001, ****P < 0.000 1. *P* values were determined using unpaired two-tailed *t*-test (**d**, **i**) or one-way ANOVA (**j**). CC Compound C

To gain insights into how the mito-condition regulated cell mitobiogenesis, we employed bulk RNA sequencing (RNA-seq). Mc-MSCs and tc-MSCs exhibited distinctive transcriptomic profiles, with 9 524 differentially expressed genes (mc-MSC/tc-MSCs, fold change ≥ 2) (Figs. 4a and S6a, b). The Gene Ontology (GO) analysis illustrated that mc-MSCs were enriched for genes involved *mRNA splicing*, *DNA replication*, *cell division*, *ribosome biogenesis*, and *mitochondrial translation* (Fig. 4b), while tc-MSCs were enriched for terms including *cell adhesion*, *extracellular matrix organization*, *cell migration*, *vesicle-mediated transport*, and *autophagy* (Fig. 4c). Notably, mc-MSCs showed significant upregulation of key genes related to mitochondrial biogenesis such as *TFAM*, *TFB2M*, *NRF1*, and *CYCS* (Fig. 4d). The Kyoto Encyclopedia of Genes and Genomes (KEGG) pathways associated with cell proliferation and metabolism (*cell cycle*, *DNA replication*, *citrate cycle (TCA cycle)*, *fatty acid metabolism*, and *oxidation phosphorylation*) were significantly enhanced in mc-MSCs (Fig. 4e). Conversely, the pathways about *lysosome*, *phagosome*, *endocytosis*, *actin cytoskeleton*, and *focal adhesion* were down-regulated (Fig. S6c). These all suggested that mc-MSCs acquired a proliferative and metabolic phenotype compared to tc-MSCs, with other cellular processes being relatively suppressed.

To further uncover the underlying mechanisms, we noticed that the AMPK pathway was impressively activated in mc-MSCs (Fig. 4e), which was well known to play a key role in sensing bioenergy demand and promoting mitochondrial biogenesis to meet cellular energy requirements.^40^ This observation was consistent with data showing that glycolysis was also promoted in mc-MSCs (Fig. S7). The up-regulation of AMPK pathway in mc-MSCs was confirmed by different characterizations. In the RNA-seq data, related genes including *PPARG*, *CCNA2*, *PPP2R3A*, *STRADB*, *PPP2CB*, and *ELAVL1* were enriched in the mito-condition group (Fig. S6d). The Gene Set Enrichment Analysis (GSEA) results further displayed significant enrichment of the AMPK pathway (Fig. 4f). To confirm the expression levels of AMPK and phosphorylated-AMPK (p-AMPK), the latter indicating activation status,^40^ we conducted western blot which showed notable elevations of both in mc-MSCs (Fig. 4g). Furthermore, we realized an upregulation of a key downstream transcription factor of AMPK, transcription factor A, mitochondrial (TFAM), which was essential for the replication and transcription of mtDNA during mitochondrial biogenesis (Fig. 4h, i).^41^ Consistent with TFAM activation, both mtDNA copy number and the expression of the mitochondrial structural protein TOMM20 were elevated in mc-MSCs compared to tc-MSCs (Figs. 4h, i and Fig. 1j), collectively suggesting a boost of mitochondrial biogenesis in mc-MSCs. Notably, we also verified the critical role of AMPK activation in the mito-condition. Treatment with the AMPK inhibitor Compound C (CC) resulted in a significant reduction of total mitochondrial intensity in mc-MSCs (Fig. 4j).

In summary, we demonstrated that the mito-condition modified cellular programs of mitobiogenesis by activating the AMPK pathway (Fig. 4k).

### The mito-condition tunes energy balance by compromising other energy-consuming processes

**Fig. 5:**
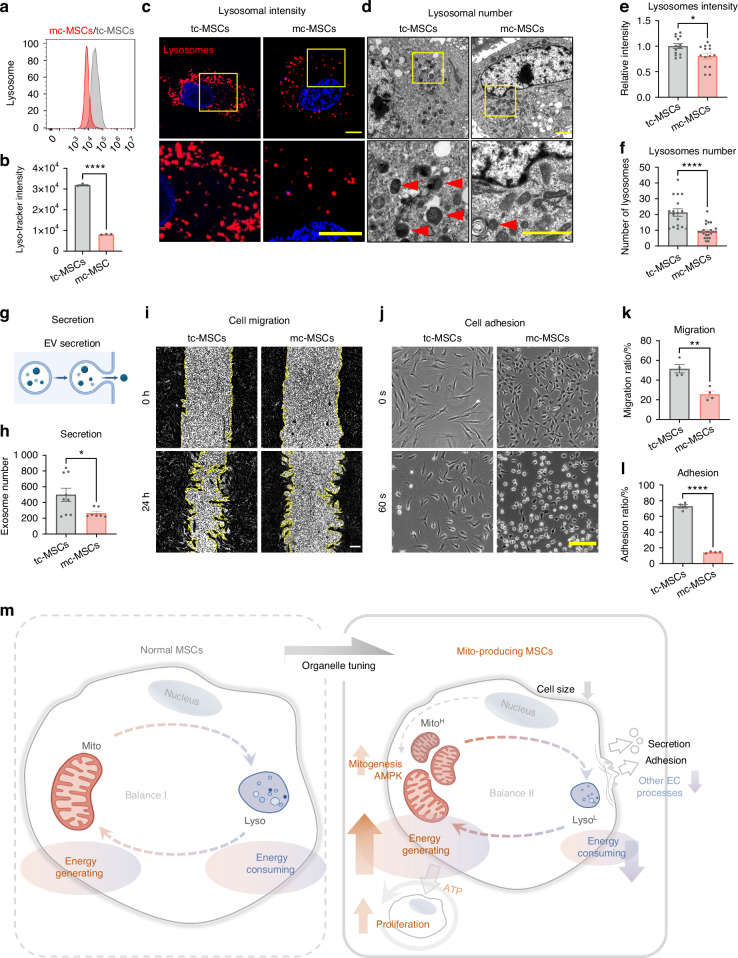
The mito-condition tunes energy balance by compromising other energy-consuming processes. **a** Flow cytometry of lyso-Tracker labeled tc-MSCs and mc-MSCs. **b** Lysosomal intensity of tc-MSCs and mc-MSCs (well = 3). **c** Representative images of lysosomes in tc-MSCs and mc-MSCs. Scale bar, 20 μm. **d** Representative transmission electron microscope (TEM) images of tc-MSCs and mc-MSCs. Scale bar, 2 μm. Red arrows: lysosomes. **e** Relative lysosomal intensity of tc-MSCs and mc-MSCs (observed cell = 14 for each group). **f** Number of lysosomes of tc-MSCs and mc-MSCs from TEM images (17 and 23 cells were calculated for tc-MSCs and mc-MSCs group respectively). **g** Schematic diagram of extracellular vesicles (EVs) secretion by MSCs. **h** Number of EVs in tc-MSCs and mc-MSCs (well = 9 in tc-MSCs group; well = 7 in mc-MSCs group). **i** Cell scratch migration assay of tc-MSCs and mc-MSCs. Scale bar, 200 μm. **j** Cell adhesion assay of tc-MSCs and mc-MSCs. Scale bar, 200 μm. **k** Migration ratio of tc-MSCs and mc-MSCs (well = 4). **l** Adhesion ratio of tc-MSCs and mc-MSCs (well = 4). **m** Schematic diagram showing the mito-condition was specialized to generate and reproduce mitochondria by increasing the energy-generating process and restricting other unnecessary energy-consuming processes. All data are presented as mean ± SEM. *P < 0.05, **P < 0.01, ****P < 0.000 1. *P* values were determined using unpaired two-tailed *t*-test (**b**, **e**, **f**, **h**, **k**, **l**)

Having uncovered the activation of the AMPK pathway, we further characterized the cellular status by comparing the down-regulated processes in mc-MSCs and tc-MSCs. Interestingly, the transcriptome data revealed that genes related to several energy-consuming cellular activities, including lysosome digestion, cell migration, adhesion, and secretion, were inhibited under the mito-condition (Fig. 4c). This indicated an overall alteration of intracellular components and programs. Therefore, we verified these finding by detecting the abundance of other organelles and their activities. Our data showed no evident changes in both the quantity and structure of the Golgi apparatus (Fig. S8). The quantity of the endoplasmic reticulum (ER) did not change significantly (Fig. S8a, b), while TEM indicated amelioration of ER swelling under the mito-condition (Fig. S8c). What’s more, we realized an obvious reduction of lysosome quantity in mc-MSCs, as demonstrated by flow cytometry (Fig. 5a, b), immunofluorescence (Fig. 5c, d) and TEM (Fig. 5e, f), which correlated with the downregulation of autophagy-related genes (Fig. 4c). Autophagy biomarkers, including Beclin1 and the LC3-II/I ratio, were significantly decreased in mc-MSCs, confirming a reduced level of autophagy and indicating a new balance of organelle activities (Fig. S9a, b). Previous studies have shown that ER swelling, a sign of ER stress, was often coupled with increased autophagy.^42,43^ This suggested that the mito-condition relieved ER stress induced by serum-containing medium, resulting in mc-MSCs exhibiting lower levels of autophagy, which might not be essential for their survival. Besides the changes in intracellular organelles, mc-MSCs demonstrated attenuation of other energy-consuming cellular activities, including extracellular vesicle secretion (Fig. 5g, h), cell migration (Fig. 5i, k) and cell adhesion (Fig. 5j, l).

Given the frequent crosstalk between mitochondria and other organelles, it was unexpected that the abundance of other structures, especially lysosomes, was reduced under the mito-condition. In other studies, lysosomal activity was often synchronized with mitochondrial function to facilitate cell catabolism and homeostasis.^44,45^ On the contrary, our data suggested that enrichment of lysosomes, levels of autophagy, and other cellular functions (migration/secretion/adhesion) were not necessarily maintained in a proliferative and energetic cell status. This further suggested that the mito-condition was specialized to generate and reproduce mitochondria by restricting other unnecessary energy-consuming processes (Fig. 5m).

### Mc-mitochondria exhibit superior performance for in vivo mitotherapy

To further demonstrate the therapeutic potential of mc-mitochondria in tissue regeneration, we applied them to OA models, a prevalent cartilage degenerative disease associated with chondrocyte mitochondrial dysfunction.^46^

Before in vivo studies, we transplanted mitochondria into human OA chondrocytes and measured their effects on chondrocyte metabolism in vitro (Fig. S10a). Results showed that both tc-Mito and mc-Mito could increase ATP production in OA chondrocytes (Fig. S10b). However, mitochondria derived from mc-MSCs induced a more pronounced improvement in ATP production and MMP in OA chondrocytes (Fig. S10c, d) and also reduced the rate of cellular apoptosis compared to mitochondria derived from tc-MSCs (Fig. S10e, f). In addition, mc-Mito transplantation effectively enhanced the expression of TFAM in chondrocytes (Fig. S10g, h), confirming that this approach could modify mitochondrial function in OA chondrocytes.

We then tested whether mitochondria could enter targeted tissues and persist in situ when they were injected into joints. Using a mouse cartilage explant model, we observed that co-cultured mitochondria attached to the cartilage surface (Fig. S11a). In vivo imaging with a DiR label to track mitochondria in mouse joints revealed fluorescence diminished by half after 7 days and completely vanished after more than 21 days (Fig. S11b, c), suggesting that these mitochondria might survive and function in vivo.

**Fig. 6:**
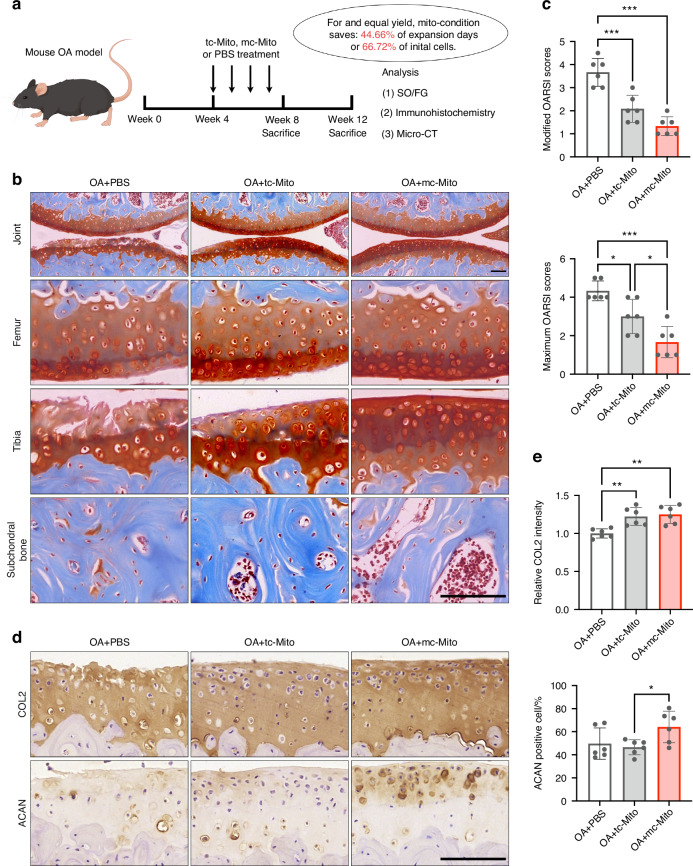
Mc-mitochondria exhibit superior performance for in vivo mitotherapy. **a** Schematic illustration of the establishment of the mouse OA model and the experimental design to evaluate the value of mitochondrial amplification for in vivo applications. **b** Safranin-O/Fast green staining of joint sections at 12 weeks. Scale bar, 50 μm. **c** Modified and maximum OARSI scoring system (sample = 6 for each group). **d** Immunohistochemical staining (COL2, ACAN) of joint sections at 12 weeks. Scale bar, 50 μm. **e** Quantification of COL2 and ACAN in cartilage tissues at 12 weeks (sample = 6 for each group). All data are presented as mean ± SEM. *P < 0.05, **P < 0.01, ***P < 0.001. *P* values were determined using one-way ANOVA (**c**, **e**). OARSI Osteoarthritis Research Society International

In the treatment of a murine medial meniscectomy (MMx) OA model, equal quantities of mc-mitochondria and tc-mitochondria were injected into murine joints (10 μg per injection, once a week), with phosphate-buffered saline (PBS) serving as a blank control (Fig. 6a). Owing to the differences in cell expansion and mitogenesis efficiency, harvesting the same amount of mitochondria using mito-condition saved 44.66% of expansion time or 66.72% of initial cells relative to the typical method (Fig. 6a). For instance, to produce 140 μg of mitochondria, the mito-condition required only 4.18 days (PDT = 20.26 h at passage 3), whereas the typical condition needed 7.49 days (PDT = 36.61 h at passage 3) starting with 2 × 10^5^ MSCs on day 1. If these mitochondria need to be fabricated within 3 days, the mito-condition strategy demanded only 5.11 × 10^5^ cells on the first day, which was 1/3 of the cell quantity required for typical culture. This further suggested that mito-condition served as an efficient platform for mitochondria fabrication.

Histological analysis of animal study results demonstrated that mitochondrial transplantation exhibited therapeutic effects on OA in both 8-week and 12-week post-surgery samples compared to the untreated group. Notably, the mc-mitochondria group showed a superior outcome in cartilage repair in the 12-week samples among the three groups (Figs. 6b, c and S12a,b). Particularly, the safranin-O staining showed that mc-mitochondria treatment resulted in a more intact cartilage surface (especially in the tibia) when compared to tc-mitochondria treatment, which was consistent with the Osteoarthritis Research Society International (OARSI) scoring evaluation (Fig. 6b, c). The immunostaining illustrated that mc-mitochondria therapy preserved the expression of cartilage functional markers collagen type II (COL2) and aggrecan (ACAN) at joint surfaces of 12-week samples (Fig. 6d, e and S12c, d). Moreover, periarticular osteophyte formation and subchondral bone sclerosis are hallmarks of OA progression. Micro-CT analysis showed that both mc-mitochondria and tc-mitochondria were capable of alleviating subchondral bone sclerosis at 8 weeks. Notably, only mc-mitochondria demonstrated the ability to reduce osteophyte formation at 8 weeks (Fig. S13).

Taken together, our animal study validated that mitochondrial transplantation could act as an effective intervention for OA. Our proposed mito-condition culture showed advantages in mitochondria production and long-term cartilage surface protection, indicating that the mito-condition could rapidly supply high-functioning mitochondria for in vivo disease management.

## Discussion

Mitochondrial transplantation serves as an encouraging therapeutic approach for various metabolic diseases.^47–49^ However, obtaining sufficient quantities of healthy human mitochondria remains a significant challenge. In this study, we proposed a novel mitochondrial fabrication strategy by identifying customized conditions that programmed stem cell mitobiogenesis and organelle balance.

We demonstrated that our live-cell mitochondria factory could robustly and sustainably fabricate off-the-shelf human mitochondria using adipose-derived MSCs, while traditional invasive methods rely on isolating mitochondria from liver or muscle tissues for one-time use. Notably, compared to typical culture conditions, our customized condition achieved a significantly higher yield within 15 days. With a dramatic improvement in cell expansion rate (a 302-fold increase) and an increase in mitochondrial quantity per cell in the mito-condition (a 2.83-fold change), the final fold change in total yield was estimated to exceed 850 (302 × 2.83 = 854). This approach potentially provides sufficient mitochondria for a substantial number of patients in future mitotherapy. Furthermore, mitochondria cultured under our conditions (mc-mitochondria) gained enhanced homeostasis in vitro and superior function in tissue repair in vivo. These findings imply that our strategy is a promising method to efficiently fabricate high-quality human mitochondria for clinical mitotherapy.

Cell engineering allows the artificial programming of cellular functions by modulating key regulatory pathways.^31^ Inspired by this concept, we developed a mitochondrion-producing condition to boost cell proliferation and mitobiogenesis. In this study, we uncovered the activation of AMPK in mc-MSCs, which serves as a highly conserved energy-sensing mechanism in energy stress and mitobiogenesis regulation.^40^ AMPK senses intracellular energy demands and stimulates the downstream effectors to promote cell proliferation and metabolism.^40^ We found that TFAM, a critical downstream target of AMPK that controlled mitochondrial transcription and replication, was significantly up-regulated in mc-MSCs (Fig. 4h). While further investigations are needed to fully elucidate the underlying mechanisms, these data partially explained the up-regulation of mitochondrial functional and structural proteins (Figs. 3g and 4h), and the rapid mitobiogenesis observed in mc-MSCs (Fig. 4k).

Interestingly, the cellular state induced by the mito-condition differed from other reported AMPK-activated states.^40^ Notably, the number of lysosomes was unexpectedly reduced in mc-MSCs, in contrast to previous findings that lysosomal activity is typically promoted in energetic cells.^50,51^ Since ATP was required for all steps of autophagy and preservation of the lysosome microenvironment, this reduction may result from decreased autophagy in the new cellular state, where cells experience lower stress levels compared to typical culture MSCs (tc-MSCs), as indicated by mitochondrial membrane potential depolarization and ER morphology (Figs. 3d and S8c). Furthermore, we observed down-regulation of other energy-consuming cellular activities, including secretion, migration, and adhesion (Fig. 5g–l). These findings supported that the mito-condition was an optimal mitobiogenesis design, as it suppressed unnecessary programs and reallocated resources toward rapid cell cycling and mitochondrial synthesis. In the absence of robust autophagy, high-speed cell division might maintain cellular homeostasis by consuming excess bioenergy and preventing mitochondrial overload in individual cells. Therefore, this artificially tuned cellular state was specialized for the generation of mitochondria, providing insights into organelle homeostasis and informing the development of new approaches in organelle therapy.

In our optimized condition, we recognized that multiple factors contributed to metabolic homeostasis. Our previous study illustrated that these factors promoted cell proliferation.^29^ We found that bFGF, Hc, Vc, and HPL were indispensable for the mito-condition, as their exclusion significantly hindered mitochondrial biosynthesis. Particularly, HPL has been shown to dramatically promote cell cycle and migration via AMPK/mTOR pathway.^34^ Additionally, Vc and bFGF have been reported to promote mitochondrial biogenesis and ATP synthesis in various cell types.^38,52,53^ Low-dose hydrocortisone (0.1 ~ 1 mmol/L) has also been shown to increase the proliferative and metabolic activity of MSCs.^39^ These findings aligned with our results and supported the necessity of these components. Although the effects of other components have not been clearly reported, our results suggested their critical roles in elevating mitobiogenesis. While further optimization is needed, we have achieved a combinatory condition that successfully promoted cell mitobiogenesis, which could aid in developing the organelle synthesis industry.

For the in vivo application, we selected OA, a predominant degenerative disease featured by metabolic impairment.^10^ Previous studies have shown the effectiveness of mitotherapy in treating various OA models,^54–56^ primarily attributed to the restoration of mitochondrial function and energy supply in affected chondrocytes.^57^ Here, we discovered that mitochondrial transplantation increased chondrocyte homeostasis, mitochondrial function, and biogenesis in vitro (Fig. S10). In our animal study, we not only confirmed the therapeutic effect of mitotherapy in OA but also demonstrated the advantages of our approach in rapid mitochondrial fabrication and joint protection (Fig. 6 and Fig. S11-13). However, we also acknowledge limitations in this study, including the lack of evidence for efficient mitochondria transfer and retention in targeted cells. Consequently, the development of advanced mitochondria delivery systems and validation of mitochondria functionality after transplantation are imperative for future research.

Overall, our organelle-tuning strategy demonstrated robust mitochondrial production under customized condition, which could be used to greatly promote in vivo tissue regeneration. This study provided new insights into the molecular regulation of intrinsic organelle biogenesis and energy balance, potentially informing the development of novel approaches in organelle therapy.

## Materials and methods

### Isolation and culture of human adipose-derived MSCs

Human samples were obtained from patients undergoing specific surgical procedures with the approval of the Ethics Committee of Second Affiliated Hospital, Zhejiang University (Approval number: 2018-037, 20230735). All sampling was performed with the patients’ informed consent. The information of donor individuals was included in Table 1. The isolation of adipose-derived MSCs was collected as previously described.^58^ In the typical condition, MSCs were cultured in low glucose Dulbecco’s modified Eagle’s medium (DMEM) (Gibco, USA) supplemented with 10% fetal bovine serum (FBS; Gibco, USA). In the mitochondrion-producing condition, MSCs were cultured in DMEM/F12 (Gibco, USA) supplemented with 5 ng/mL bFGF (100-18B, Peprotech, USA), 1.722 g/L NaHCO_3_ (25080-094, Gibco, USA), 0.1% lipid concentrate (11905-031, Gibco, USA), 1× ITS (41400-045, Gibco, USA), 17.8 nmol/L Prog (S1705, Selleck, USA), 100 nmol/L Hc (S1696, Selleck, USA), 197.6 μmol/L Vc (A8960, Sigma, USA), 10 μg/mL HS (S1346, Selleck, USA) and 1% HPL (PLTGOLD100R, Biological Industries, Israel). Human chondrocytes were isolated as previously described.^59^ Chondrocytes were cultured in DMEM/F12 with 10% FBS.

**Table 1:** Human samples information

Individual	Age/year	Gender	Disease	Sample location	Figures
Human adipose-derived stem cells	56	M	Fracture	Thigh fat	Fig. 2a(#1), 2b, 2c and 2k
	64	M	Osteoarthritis (joint replacement)	Thigh fat	Fig. 2a(#2), 2b, 2c and 2k
	72	F	Osteoarthritis (joint replacement)	Thigh fat	Fig. 2a(#3), 2b, 2c and 2k
	26	M	Fracture	Thigh fat	Figs. 1c, 1d, S1, and S3
	10	M	Fracture	Thigh fat	Figs. 1e-j, 2d-j, 3, 4, 5, S2 and S4-9
Human osteoarthritis chondrocytes	64	F	Osteoarthritis (joint replacement)	Osteoarthritis cartilage	Fig. S10

### Cell viability and cell proliferation assay

Cells were seeded into 96-well plates at a density of 3 000 cells/cm^2^ and incubated for 3 days in the typical condition and mito-condition, then cell viability was measured using a Cell Counting Kit-8 (CCK-8, Dojindo, Japan) assay following the manufacturer’s instructions. Briefly, the CCK-8 solution was added into the culture medium at 1:10. Absorbance at 450 nm was measured after 2 h. The PDT of the cells was calculated using the following formula: PDT = culture time ∗ log_2_/log(final cell numbers/initial cell numbers). Cell cycle assay was measured using the cell cycle and apoptosis analysis kit (C1052, Beyotime, China). The EdU proliferation assay was performed using the BeyoClick™ EdU cell proliferation kit (C0071, Beyotime, China).

### Ultrastructure visualization

For TEM observation, ~2 × 10^6^ cells were centrifuged to form pellets and fixed with 2.5% glutaraldehyde at 4°C for 24 h. Then, the cells were rinsed 3 times in PBS for 10 min each and postfixed in 1% osmium acid solution for 1 h. After 3 washes with PBS for 15 min each, the cells were stained by 2% aqueous uranyl acetate for 30 min and dehydrated in a graded ethanol series (50%, 70%, 90%, 100%) and acetone (100%, 100%) for 20 min each and then embedded in a 100% Epon. Thin sections were stained with lead citrate and visualized using Philips Tecnai 10 TEM (Philips, Netherlands).

### mtDNA copy number assay

Cell genomic DNA was extracted using DNeasy Blood & Tissue Kit (69504, Qiagen, USA). The mtDNA content was determined as the ratio of the copy number of mtDNA to the copy number of nuclear DNA using the human mtDNA monitoring primer set (7246, Takara, Japan).

### Immunofluorescence staining

Cells were fixed in 4% polyformaldehyde (PFA) for 20 min and then incubated in 0.3% Triton X-100 for 10 min and blocked with 1% bovine serum albumin (BSA; Sangon, China) for 30 min at RT. Afterwards, samples were incubated with primary antibodies at 4°C overnight and then with appropriate fluorescent probe-conjugated secondary antibodies for 2 h at RT. For organelle staining, cells were stained with Mito-Tracker Red CMXRos (C1035, Beyotime, China) and Lyso-Tracker Red (C1046, Beyotime, China). Cell nuclei were stained with 4′,6-Diamidino-2-phenylindole (DAPI; C1002, Beyotime, China). Imaging was performed with an Olympus FV1000 confocal microscope and analyzed by Fiji or Imaris software. The antibodies were listed in Table 2.

**Table 2:** Antibody List

Name	Source	Application
CD34-APC	Biolegend, 343607	1:20 (FC)
CD45-PE	Biolegend, 368510	1:20 (FC)
CD73-PE	Biolegend, 344003	1:20 (FC)
CD90-APC	Biolegend, 328114	1:20 (FC)
CD105-PE	eBioscience, 12-1057-42	1:20 (FC)
Mouse anti-TOMM20	Abcam, ab283317	1:500 (IF)
Rabbit anti-Ki67	Abcam, ab16667	1:250 (IF)
Rabbit anti-GM130	Proteintech, 11308-1-AP	1:200 (IF)
Rabbit anti-Calnexin	Proteintech, 10427-2-AP	1:200 (IF)
Rabbit anti-AMPK	Abcam, ab32047	1:3 000 (WB)
Rabbit anti-phospho-AMPK	Cell Signaling Technology, 2535	1:1 000 (WB)
Rabbit anti-ATP5A1	Proteintech, 14676-1-AP	1:2 000 (WB)
Rabbit anti-UQCRC1	Proteintech, 21705-1-AP	1:2 000 (WB)
Rabbit anti-SDHB	Proteintech, 10620-1-AP	1:2 000 (WB)
Rabbit anti-MTCO2	Proteintech, 55070-1-AP	1:2 000 (WB)
Rabbit anti-TFAM	Proteintech, 22586-1-AP	1:5 000 (WB)
Rabbit anti-TOMM20	Proteintech, 11802-1-AP	1:5 000 (WB)
Rabbit anti-LC3	Proteintech, 81004-1-RR	1:2 000 (WB)
Mouse anti-β-Actin	Proteintech, 66009-1-Ig	1:5 000 (WB)
Mouse anti-GAPDH	Proteintech, 60004-1-Ig	1:5000 (WB)
Mouse anti-COL2	Santa cruz, sc-52658	1:50 (IHC)
Rabbit anti-ACAN	Abcam, ab36861	1:200 (IHC)
Acti-stain™ 488 phalloidin	Cytoskeleton, PHDG-1	1:500 (IF)
Goat anti-Rabbit 488	Invitrogen, A11008	1:500 (IF)
Donkey anti-Mouse 488	Invitrogen, A21202	1:500 (IF)
Goat anti-Rabbit HRP	Jackson, 111-035-003	1:500 (IHC); 1:3 000 (WB)
Goat anti-Mouse HRP	Jackson, 115-035-003	1:500 (IHC); 1:3 000 (WB)

FC, flow cytometry; IF, immunofluorescence; IHC, immunohistochemistry; WB, western blot

### Flow cytometry analysis

Cells were harvested and blocked with 1% BSA for 30 min at 4°C. Subsequently, the cells were stained with fluorescein-conjugated antibodies for 30 min at 4°C. The corresponding isotype antibodies were used as controls. For organelle staining, cells were stained with Lyso-Tracker Red (C1046, Beyotime, China), Golgi-Tracker Red (C1043, Beyotime, China), and ER-Tracker Green (C1042, Beyotime, China) for 20 min at 37°C. The assay was performed by DxFLEX (Backman Coulter, USA) and analyzed using FlowJo software. The antibodies were listed in Table 2.

### Trilineage Differentiation

For osteogenic differentiation, cells were seeded at a density of 1 × 10^4^ cells/cm^2^. After 24 h, the culture media were replaced with osteogenic differentiation media, which comprised high glucose DMEM (H-DMEM) supplemented with 10% FBS, 0.1 μmol/L dexamethasone, 50 μmol/L Vc, 10 mmol/L β-glycerol phosphate. After 21 days, the cells were fixed in 95% ethanol and stained with 2% alizarin red solution. The stained calcium-rich extracellular matrix was visualized under an inverted microscope (IX73, Olympus, USA). For quantitative analysis, 5% sodium dodecyl sulfonate (SDS; SB0485, Bio Basic, Canada) with hydrochloric acid (SDS/HCl) was added into the well and incubated for 30 min. Supernatants were used to obtain optical density (OD) values of the dye by measuring the absorbance at 405 nm.

For chondrogenic differentiation, cells were seeded at a density of 5 × 10^4^ cells/cm^2^. After 24 h, the culture media were replaced with chondrogenic differentiation media, which comprised H-DMEM supplemented with 1% sodium pyruvate, 1% ITS, 100 nmol/L dexamethasone, 50 μg/mL Vc and 10 ng/mL TGF-β3. After 21 days, the production of glycosaminoglycan (GAG) was measured by alcian blue staining and visualized under an inverted microscope. For the quantitative analysis of alcian blue staining, the staining intensity was measured and analyzed using ImageJ.

For adipogenic differentiation, cells were seeded at a density of 5 × 10^4^ cells/cm^2^. After 24 h, the culture medium was replaced with adipogenic differentiation media, which comprised H-DMEM supplemented with 10% FBS, 1 μmol/L dexamethasone, 500 μmol/L 3-isobutyl-1-methylxanthine, and 200 μmol/L indomethacin. After 14 days, the cells were fixed in 4% PFA for 30 min. Lipid droplets of the differentiated cells were stained by oil red. The stained samples were visualized using an inverted microscope. For quantitative analysis, 100% isopropanol was added into the well and incubated for 30 min. The supernatant was transferred to obtain the OD value of the dye by measuring its absorbance at 510 nm.

### Colony formation unit (CFU) assay

Cells were seeded into 6-well plates at a density of ~200 cells per well followed by culturing for 14 days. Cells were fixed with 4% PFA and stained with 0.1% crystal violet methanol solution at RT for 20 min. After removing the float dyestuff, the clone formation was observed under the microscope.

### Isolation and transplantation of mitochondria

The isolation of mitochondria was achieved using the cell mitochondria isolation kit (C3601, Beyotime, China) combined with a glass homogenizer. Cell pellets were suspended into 1 mL of ice-cold lysis buffer for 15 min incubation followed by homogenate by 24 strokes. The large cell debris was separated by centrifugation at 600 × *g* for 10 min at 4°C, and the supernatant was further centrifuged at 11 000 × *g* for 10 min at 4°C to collect the mitochondrial pellets. The mitochondrial pellets were rinsed with 1 mL mitochondria storage buffer and isolated by another centrifugation at 11 000 × *g* for 10 min at 4°C. Isolated mitochondria were suspended with PBS and applied freshly for the subsequent experiments. Isolated mitochondria were transplanted to MSCs by coculture^60^ or transplanted to chondrocytes by centrifugation^61^ using published protocols.

### Live-cell metabolic assay

The oxygen consumption rate (OCR) and extracellular acidification rate (ECAR) values were measured with a Seahorse XF96 extracellular flux analyzer (Agilent, USA). Briefly, cells were seeded in an XF96 microplate at a density of 10^4^ cells per well and preincubated overnight. Before the assays, cells were equilibrated for 1 h in unbuffered XF assay medium (for OCR: base medium with 10 mmol/L glucose, 2 mmol/L glutamine, and 1 mmol/L pyruvate, pH 7.4; for ECAR: base medium with 1 mmol/L glutamine). The XF Cell Mito Stress Test kit and XF Glycolysis Stress Test kit were used following standard protocols. During the assay, compounds were injected at the following final concentrations: 1.5 μmol/L oligomycin, 1 μmol/L carbonyl cyanide 4-(trifluoromethoxy)phenylhydrazone (FCCP), and 0.5 μmol/L rotenone/antimycin A(AA) for OCR or 10 mmol/L glucose, 1 mmol/L oligomycin and 50 mmol/L 2-2-deoxyglucose (2-DG) for ECAR. All Seahorse measures were normalized by the number of cells counted in each well at the end of the Seahorse experiments.

Seahorse assays also be performed on isolated mitochondria. Mitochondrial assay solution (MAS) was composed of 70 mmol/L sucrose, 220 mmol/L mannitol, 5 mmol/L KH_2_PO_4_, 5 mmol/L MgCl_2_, 2 mmol/L HEPES, 1 mmol/L EGTA, and 0.2% (w/v) fatty acid-free BSA, pH 7.2 at 37°C. 25 μL isolated mitochondria were suspended in MAS and loaded at a density of 6 μg/well into the XF 96-well cell culture microplate by centrifugation at 2 000 ×g for 20 min at 4°C. After centrifugation, 155 μL of prewarmed (37°C) MAS + substrate (10 μmol/L succinic acid and 10 μmol/L malic acid) was added to each well. During the assay, compounds were injected at the following final concentrations: 4 mmol/L adenosine 5′-diphosphate (ADP), 2.5 μg/mL oligomycin, 4 μmol/L FCCP, and 4 μmol/L AA for OCR.

### ATP assay, JC-1 assay, ROS assay, and apoptosis assay

The ATP concentration of MSCs/chondrocytes and isolated mitochondria was measured using the enhanced ATP assay kit (S0027, Beyotime, China) according to the manufacturer’s instructions. MMP of MSCs/chondrocytes and isolated mitochondria was measured using the enhanced MMP assay kit with JC-1 (C2003, Beyotime, China) according to the manufacturer’s instructions. Total intracellular ROS levels in MSCs were detected by a ROS assay kit (S0033, Beyotime, China) based on the standard protocol. The detection of MMP of isolated mitochondria was measured using a Varioskan Flash luminometer (ThermoFisher Scientific, USA). Apoptosis of chondrocytes was assessed using an Annexin V-FITC apoptosis detection kit (C1062, Beyotime, China) by flow cytometry, following the manufacturer’s instructions. Flow cytometry was performed by DxFLEX (Backman Coulter, USA) and analyzed using FlowJo software.

### Real-time quantitative PCR

Total RNA of MSCs was extracted using RNAiso Plus (9109, Takara, Japan), and cDNA was obtained using a reverse transcription kit (Toyobo, Japan). The RNA levels were quantified by real-time PCR with TB Green Premix Ex Taq (TaKaRa, Japan). Fluorescence quantification analysis was carried out by a high throughput fluorescent quantitative PCR instrument (Roche, Deutschland). The primers were shown in Table 3.

**Table 3:** Primer sequences for RT-qPCR

Primer Name	Base sequence
Human-ATP5A1-F	GTATTGCCCGCGTACATGG
Human-ATP5A1-R	AGGACATACCCTTTAAGCCTGA
Human-UQCRC1-F	GGGGCACAAGTGCTATTGC
Human-UQCRC1-R	GTTGTCCAGCAGGCTAACC
Human-SDHB-F	ACAGCTCCCCGTATCAAGAAA
Human-SDHB-R	GCATGATCTTCGGAAGGTCAA
Human-GAPDH-F	TGACGCTGGGGCTGGCATTG
Human-GAPDH-R	GGCTGGTGGTCCAGGGGTCT

### Bulk RNA-seq analysis

The total RNA of MSCs was extracted using RNAiso Plus (9109, Takara, Japan) according to the manufacturer’s instructions. Library construction and sequencing were conducted using BGISEQ-500 platform (BGI, China). Differentially expressed genes (DEGs) with adjusted *P* values < 0.05 and fold change ≥ 2 were selected for further analyses. Bioinformatics analysis, including heatmap clustering, principal component analysis (PCA), GO analysis, KEGG pathway analysis, and GSEA analysis was also carried out with the online platform Dr. Tom (BGI Company, China)

### Western blot analysis

Cells were lysed with lysis buffer (P0013, Beyotime, China), and protein concentrations were determined by the enhanced BCA protein assay kit (P0010, Beyotime, China). Protein samples were separated by 10% sodium dodecyl sulfate-polyacrylamide gel electrophoresis (SDS-PAGE) and transferred onto a polyvinylidene difluoride (PVDF) membrane (Millipore, USA). The membranes were blocked in 5% BSA at RT for 1 h and then incubated with primary antibodies at 4°C overnight. Later, the membranes were washed three times with Tris-buffered saline with Tween 20 (TBST) and continued to be incubated with the secondary antibodies at RT for 2 h. Finally, the membranes were developed using an enhanced chemiluminescence (ECL) kit (P0018, Beyotime, China). The antibodies were listed in Table 2.

### Cell scratch migration assay

A scratch migration assay was performed when cell density reached ~100%. Floating cells were cleaned with PBS washing and serum-free DMEM was applied to each well for 24 h. The ratio of cell migration was calculated using Fiji software.

### Cell adhesion assay

Cells were seeded into 6-well plates at a density of 3 000 cells/cm^2^ and incubated for 3 days. Then 1% trypsin was added to the well and the cell dissociation was observed by the microscope and analyzed using Fiji software.

### Isolation of extracellular vesicles (EVs)

The isolation of EVs from MSCs was performed as previously described.^62^ EVs quantification was assessed with the NanoSight NS500 system (Malvern, UK).

### Animal experiments

All animal experiments were approved by the Laboratory Animal Welfare and Ethics Committee of Zhejiang University (ZJU20220411). C57BL/6 mice (8-week-old, bodyweight 20-25 g, purchased from Shanghai Laboratory Animal Company) were used in this study. The MMx OA model was performed as previously described.^63^ Briefly, a 1 cm longitudinal incision was made along the distal patellar tendon. The medial meniscus was exposed and then removed using surgical scissors along the lower edge of the meniscus. Then, the medial capsular incision was sutured and the skin was closed. All surgical procedures followed the aseptic principle. At 4 weeks after surgery, intra-articular injections were performed once a week for 4 weeks. Mice were randomly divided into three groups: sterilized PBS group, tc-mitochondria (10 μg tc-mitochondria suspended in 10 μL PBS) group, and mc-mitochondria (10 μg mc-mitochondria suspended in 10 μL PBS) group. The mice were sacrificed at 8 weeks and 12 weeks after the surgery and the knee joints were collected for further experiments.

### Micro-computed tomography (micro-CT) analysis

The knee joint samples were fixed with 4% PFA and were scanned using a high-resolution micro-CT scanner U-CT-XUHR (Milabs, Netherlands). The bone volume (BV) of calcified meniscus and synovium, as well as trabecular separation (Tb.Sp) of subchondral bone, was measured using Imalytics Preclinical software (Philips, Germany).

### Histological analysis and immunohistochemistry

The knee joint samples were fixed with 4% PFA for 48 h and subsequently decalcified in 10% ethylenediaminetetraacetic acid disodium salt (EDTA) solution for 2 weeks. Then the samples were paraffin-embedded and cut into 5 μm thick sections. Safranin-O and fast green (SO/FG) staining were carried out following standard protocols. Histological scoring based on the OARSI grading system (grades 0–6)^64^ was performed, and the average and maximum of the scores were calculated. Immunohistochemistry was performed to assess COL2 and ACAN levels. After gradient dewaxing, paraffin sections were soaked in the sodium citrate buffer solution at 65°C overnight for antigen retrieval. The sections were permeabilized with 0.1% Triton X-100 followed by blockade with 3% H_2_O_2_. Next, the sections were blocked with 5% BSA, and then incubated with the primary antibodies overnight at 4°C. Corresponding horseradish peroxidase- (HRP-) conjugated secondary antibodies were used for 2 h at RT. The staining was visualized with diaminobenzidine (DAB; DA1010, Solarbio, China) and co-stained with hematoxylin. A slide scanner (VS200, Olympus, Japan) was used to acquire digital micrographs. Semi-quantification analysis of immunohistochemical staining intensity of COL2 was performed by ImageJ.^65^ The antibodies were listed in Table 2.

### Bioluminescence imaging (BLI)

DiR-labeled mitochondria were intra-articular injected into the mice’s knee joints. For in vivo luminescence imaging, mice were anesthetized with isoflurane and measured by the IVIS Spectrum imaging system (Perkin Elmer, USA).

### Statistical analysis

Data were tested for homogeneity of variances and normality. Homogeneity of variances was estimated with the Levene test. Data normality was tested using Shapiro–Wilk normality test and the D’Agostino-Pearson omnibus test. The data were presented as mean ± standard error of the mean (SEM). Unpaired two-tailed Student’s t-test was used for comparisons between the two groups. Comparisons between multiple groups were performed by one-way analysis of variance (ANOVA) with the post-hoc Tukey test. Statistical significance is denoted as below: ns (indicating no statistical significance), *P < 0.05, **P < 0.01, ***P < 0.001, ****P < 0.000 1. Statistical analysis was performed using GraphPad Prism 9.

## Supplementary information

Supplementary Materials

## Data Availability

All data and material in this study are available upon request.
