# Supplementary material for: Organelle-tuning condition robustly fabricates energetic mitochondria for cartilage regeneration
Source: Bone Res. 2025 Mar 17;13:37. doi: 10.1038/s41413-025-00411-6 (PMC11914688; doi:10.1038/s41413-025-00411-6)
Supplement: Supplementary file 1 — Supplementary Materials [file 41413_2025_411_MOESM1_ESM.docx]

**Supplementary Materials for**

**Organelle-tuning condition robustly fabricates energetic mitochondria for cartilage regeneration**

**Supplemental figures and tables**

Fig. S1 A screen-defined condition efficiently increases mitochondrial intensity.

Fig. S2 Mc-MSCs display higher proliferative ability than tc-MSCs.

Fig. S3 Mc-MSCs display typical surface markers and higher stemness.

Fig. S4 Quantification of OXPHOS-related proteins (ATP5A1, UQCRC1, SDHB, and MTCO2) expression in tc-MSCs and mc-MSCs.

Fig. S5 Characterization of isolated mitochondria.

Fig. S6 Overall transcriptome analysis of mc-MSCs.

Fig. S7 Mc-MSCs show higher glycolytic activity than tc-MSCs.

Fig. S8 The structure and content of the Golgi apparatus and endoplasmic reticulum (ER) in tc-MSCs and mc-MSCs.

Fig. S9 Mc-MSCs display lower autophagy levels than tc-MSCs.

Fig. S10 Mc-mitochondria enhance the cellular and mitochondrial function of human osteoarthritis chondrocytes.

Fig. S11 *In vivo* retention time of mitochondrial transplantation.

Fig. S12 Mc-mitochondria exhibit superior performance for *in vivo* mitotherapy at 8 weeks.

Fig. S13 Mc-mitochondria treatment alleviates subchondral bone sclerosis and decreases periarticular osteophyte formation.

Table 1. Human samples information.

Table 2. Antibody List.

Table 3. Primer sequences for RT-PCR.

**
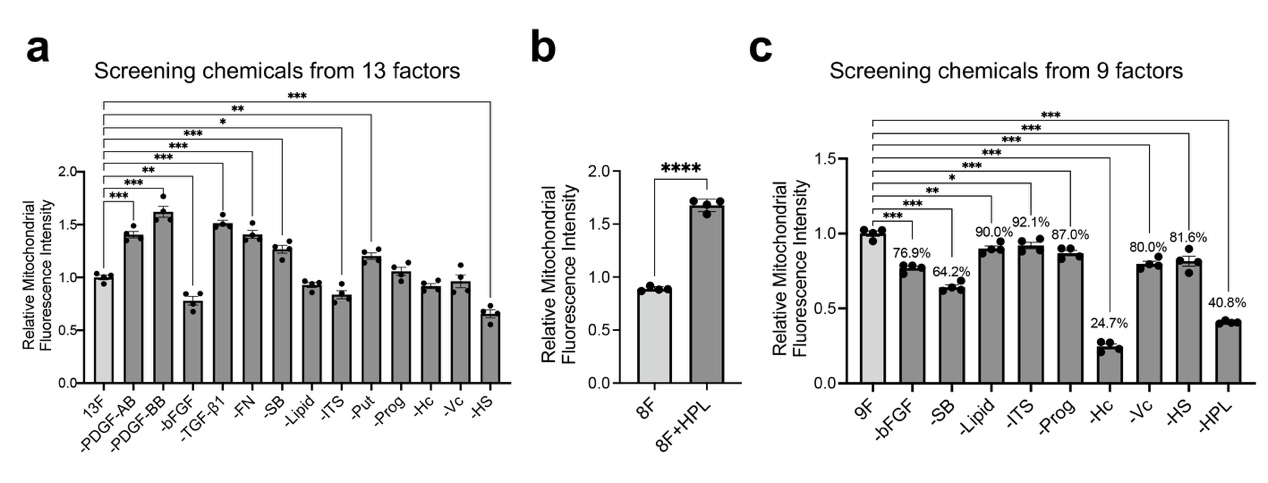
**

**Fig. S1** A screen-defined condition efficiently increases mitochondrial intensity. **a** The relative mitochondrial intensity of MSCs after withdrawing individual chemicals from 13 factors (well = 4). **b** The relative mitochondrial intensity of MSCs after adding HPL into 8 factors (well = 4). **c** The relative mitochondrial intensity of MSCs after withdrawing individual chemicals from 9 factors (well = 4). All data are presented as mean ± SEM. *p < 0.05, **p < 0.01, ***p < 0.001, ****p < 0.0001. P values were determined using unpaired two-tailed t-test (**b**) or one-way ANOVA (**a**,**c**). PDGF-AB platelet-derived growth factor-AB, PDGF-BB platelet-derived growth factor-BB, bFGF basic fibroblast growth factor, TGF-β1 transforming growth factor-β1, FN fibronectin, SB sodium bicarbonate, Lipid lipid concentrate, ITS Insulin-Transferrin-Selenium, Put putrescine, Prog progesterone, Hc hydrocortisone, Vc Vitamin C, HS heparin sodium, HPL human platelet lysate.


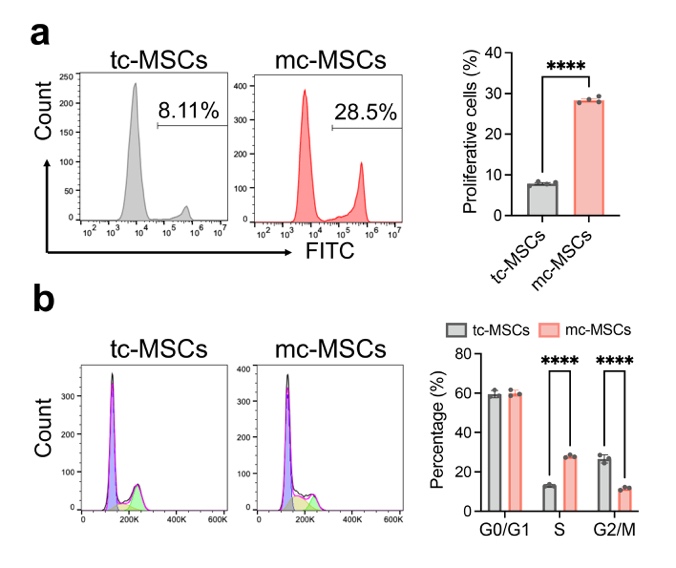


**Fig. S2** Mc-MSCs display higher proliferative ability than tc-MSCs. **a** Quantification of EdU positive cells using flow cytometry (well = 4). **b** Flow cytometric analysis of cell cycle (well = 3). All data are presented as mean ± SEM. ****p < 0.0001. P values were determined using unpaired two-tailed t-test (**a**,**b**).


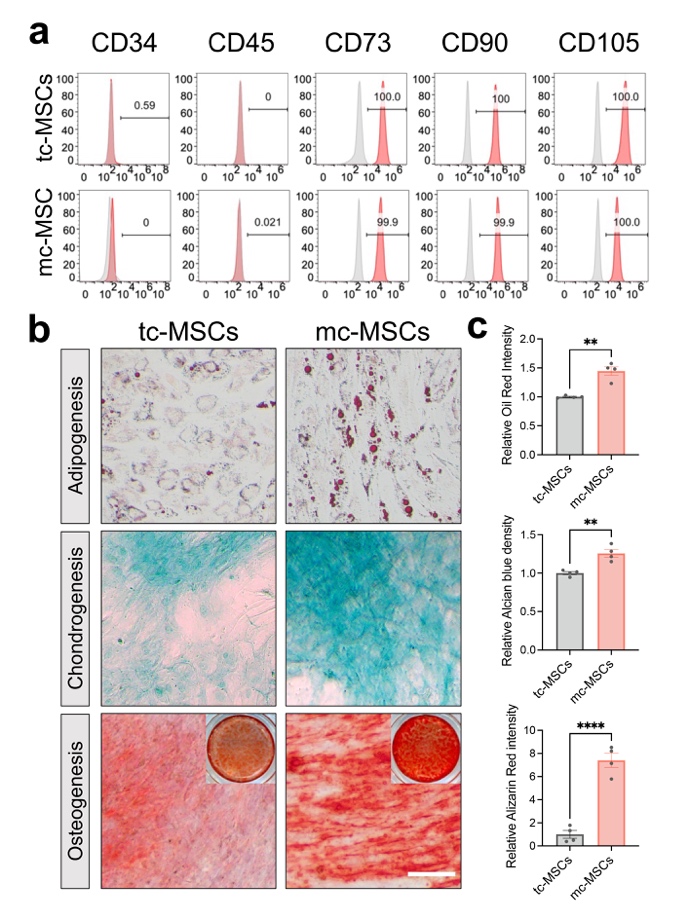


**Fig. S3** Mc-MSCs display typical surface markers and higher muti-lineage differentiation potential. **a** Flow cytometry of common MSCs surface markers (CD34, CD45, CD73, CD90, and CD105). **b** Representative images of adipogenic differentiation**,** osteogenic differentiation and chondrogenic differentiation of tc-MSCs and mc-MSCs. Scale bar, 100 μm. **c** Quantitative analysis of staining results of (**b**) (well = 4 for each group). All data are presented as mean ± SEM. **p < 0.01, ****p < 0.0001. P values were determined using unpaired two-tailed t-test (**c**).

**Fig. S4** Quantification of OXPHOS-related proteins (ATP5A1, UQCRC1, SDHB, and MTCO2) expression in tc-MSCs and mc-MSCs (well = 3). All data are presented as mean ± SEM. *p < 0.05, **p < 0.01, ***p < 0.001. P values were determined using unpaired two-tailed t-test.


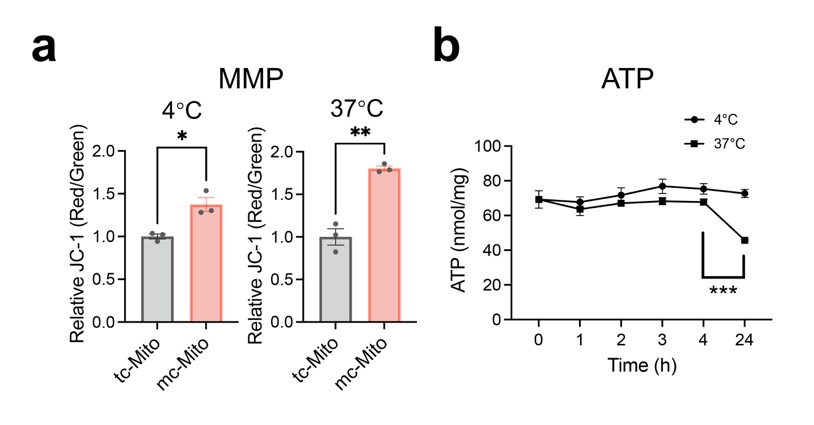


**Fig. S5** Characterization of isolated mitochondria. **a** Quantification of the JC-1 fluorescence ratio of tc-Mito and mc-Mito stored in PBS for 24 hours at 4℃ or 37℃ (well = 3). **b** change during the storage of isolated mc-Mito at 4℃ or 37℃ (well = 3). All data are presented as mean ± SEM. *p < 0.05, **p < 0.01, ***p < 0.001. P values were determined using unpaired two-tailed t-test (**a**) or one-way ANOVA (**b**).


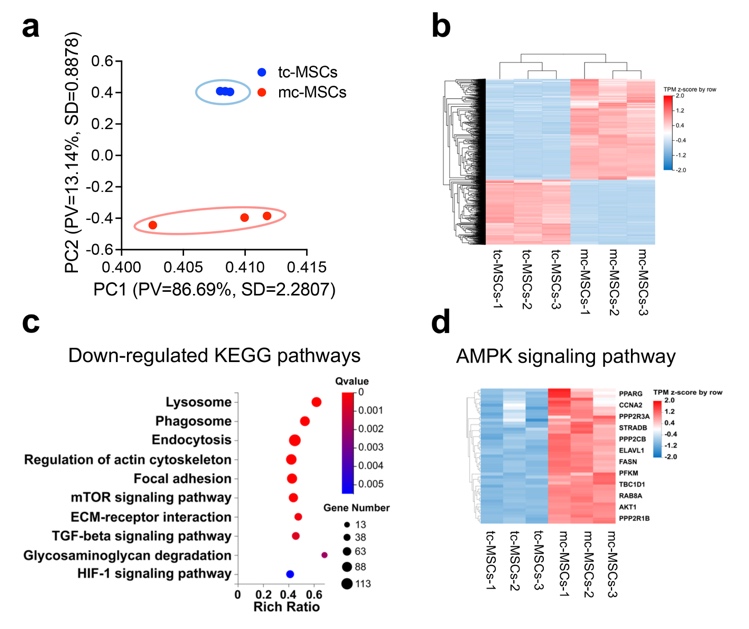


**Fig. S6** Overall transcriptome analysis of mc-MSCs and tc-MSCs. **a** PCA plot of tc-MSCs and mc-MSCs. **b** Heatmap of DEGs between tc-MSCs and mc-MSCs. **c** Downregulated KEGG pathways. **d** Heatmap of AMPK signaling pathway.


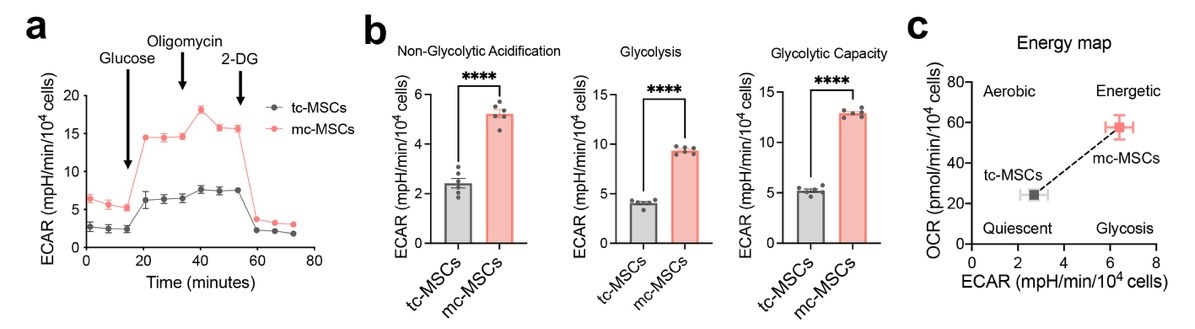


**Fig. S7** Mc-MSCs show higher glycolytic activity than tc-MSCs. **a** ECAR analysis of tc-MSCs and mc-MSCs (well = 6). **b** Quantitative analysis of ECAR results (well = 6). **c** Two-dimensional bioenergetic profiles of tc-MSCs and mc-MSCs (well = 6). All data are presented as mean ± SEM. ****p < 0.0001. P values were determined using unpaired two-tailed t-test (**b**).


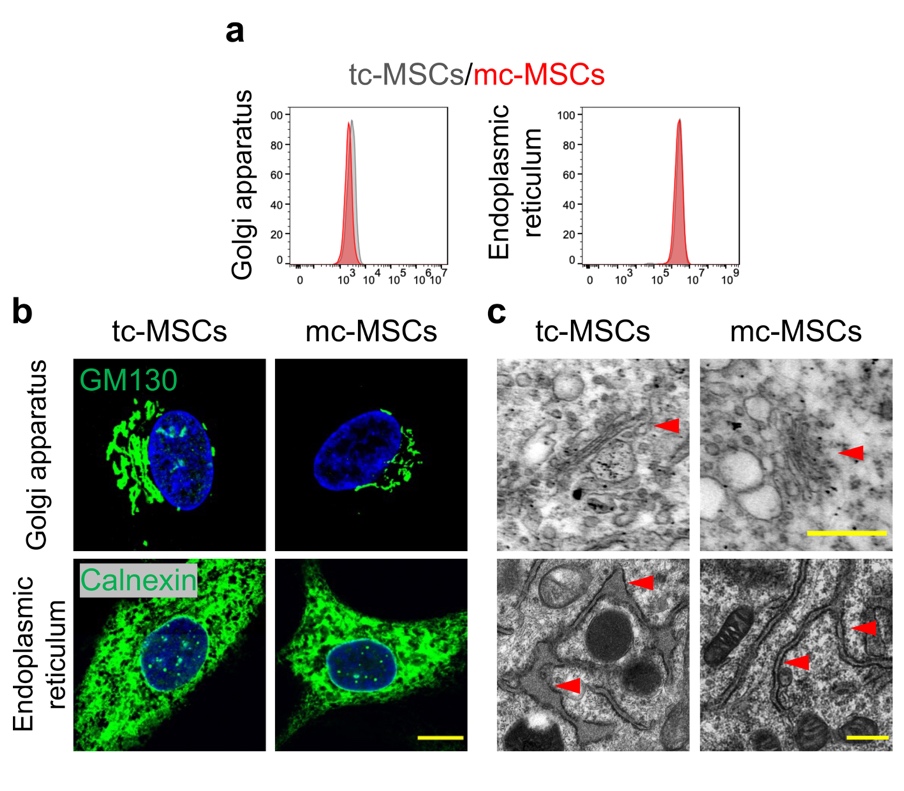


**Fig. S8** The structure and content of the Golgi apparatus and endoplasmic reticulum (ER) in tc-MSCs and mc-MSCs. **a** Flow cytometry of Golgi-Tracker or ER-Tracker labeled tc-MSCs and mc-MSCs. **b** Representative immunofluorescence images of Golgi apparatus (GM130 positive) and ER (Calnexin positive) in tc-MSCs and mc-MSCs. Scale bar, 10 μm. **c** Representative transmission electron microscope (TEM) images of Golgi apparatus and ER of tc-MSCs and mc-MSCs. Scale bar, 0.5 μm. Red arrows: Golgi apparatus or ER.


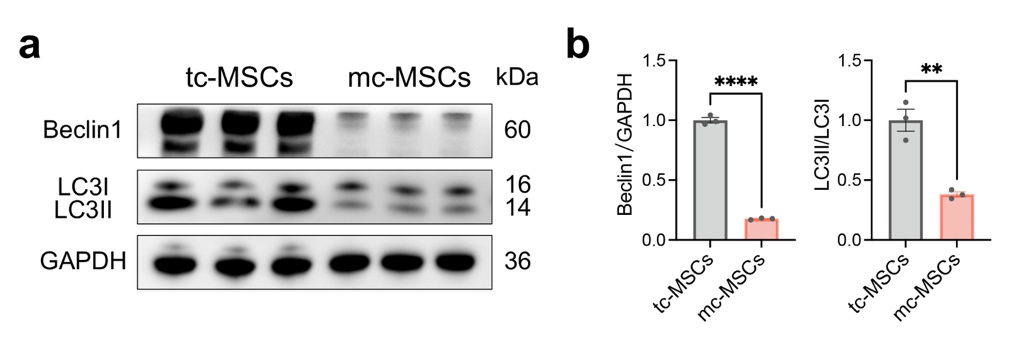


**Fig. S9** Mc-MSCs display lower autophagy level than tc-MSCs. **a** WB analysis of Beclin1 and LC3 expression in tc-MSCs and mc-MSCs. **b** Quantification of WB results in (**a**) (well = 3). All data are presented as mean ± SEM. **p < 0.01, ****p < 0.0001. P values were determined using unpaired two-tailed t-test (**b**).


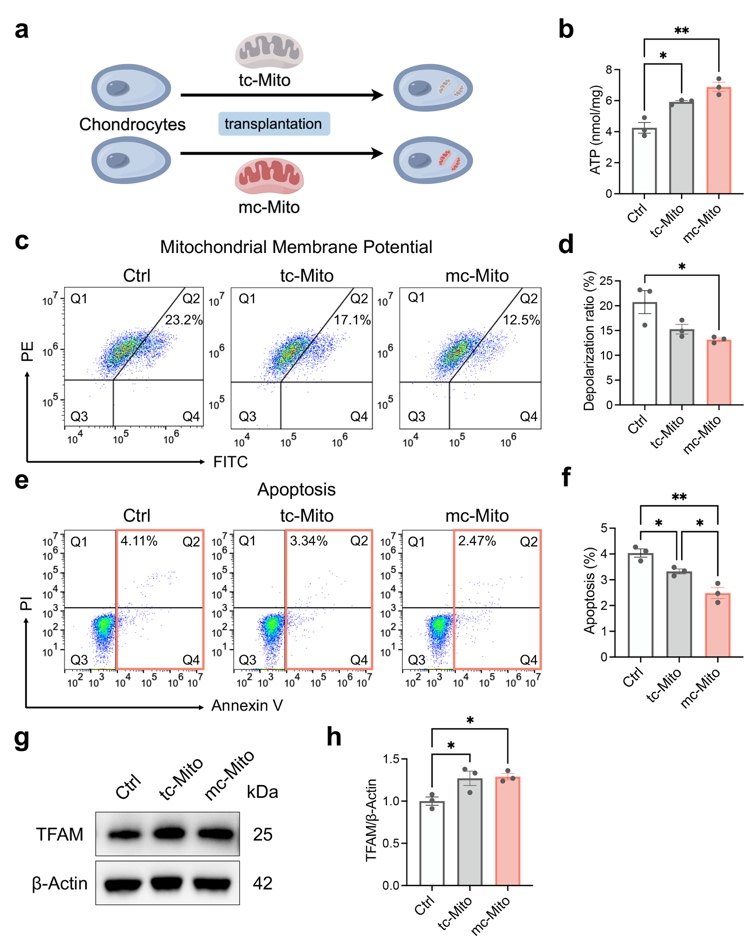


**Fig. S10** Mc-mitochondria enhance the cellular and mitochondrial function of human osteoarthritis chondrocytes. **a** Schematic illustration of the transplantation of tc-Mito and mc-Mito into human osteoarthritis (OA) chondrocytes. **b** ATP content of OA chondrocytes after mitochondrial transplantation (well = 3). **c** Representative images of flow cytometry analysis of mitochondrial membrane potential (MMP) of OA chondrocytes after mitochondrial transplantation. **d** Quantification of MMP analysis in (**c**) (well = 3). **e** Representative images of flow cytometry analysis of OA chondrocytes apoptosis after mitochondrial transplantation. **f** Quantification of apoptosis ratio in (**e**) (well = 3). **g** WB analysis of TFAM expression in OA chondrocytes after mitochondrial transplantation. **h** Quantification of WB results in (**g**) (well = 3). All data are presented as mean ± SEM. *p < 0.05, **p < 0.01. P values were determined using one-way ANOVA (**b**,**d**,**f**,**h**).


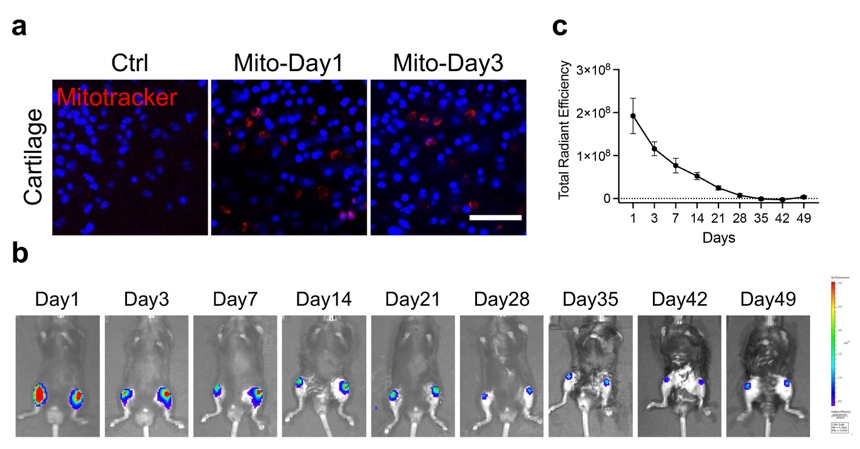


**Fig. S11** *In vivo* retention time of mitochondrial transplantation. **a** Immunofluorescence of mitochondria on cartilage explant. Scale bar, 40 μm. **b** *In vivo* bioluminescence imaging (BLI) of mice at 1, 3, 7, 14, 21, 28, 35, 42, and 49 days after injection of mitochondria. **c** Quantification of BLI results (sample =10).


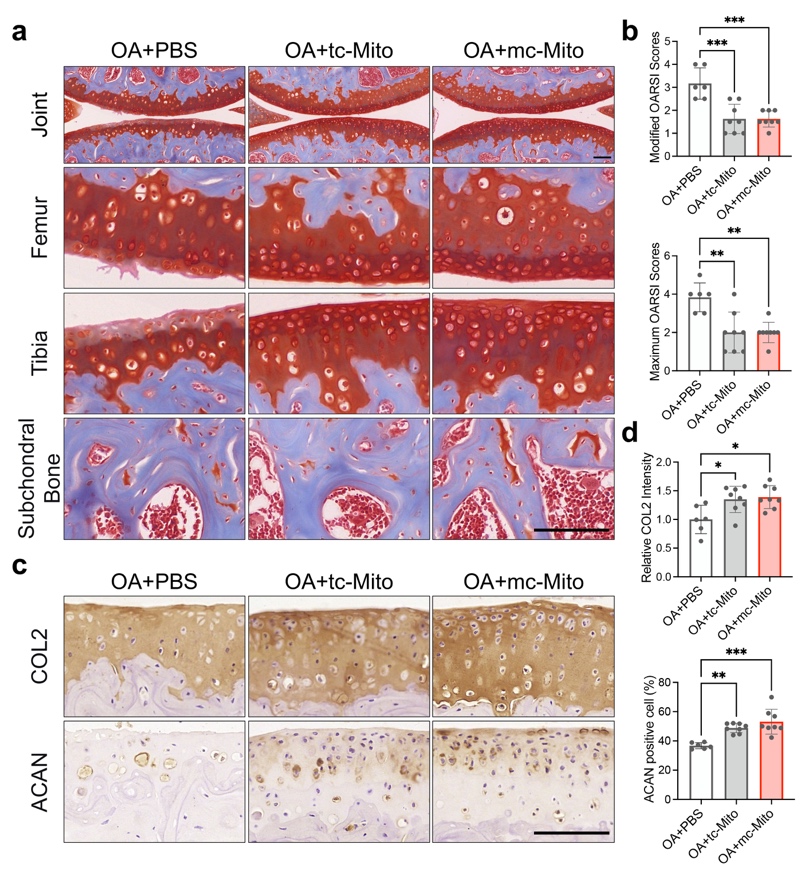


**Fig. S12** Mc-mitochondria exhibit superior performance for *in vivo* mitotherapy at 8 weeks. **a** Safranin-O/Fast green staining of joint sections at 8 weeks. Scale bar, 100 μm. **b** Modified and maximum OARSI scoring system (sample = 6 for OA+PBS group, sample = 8 for OA+tc-Mito group and OA+mc-Mito group). **c** Immunohistochemical staining (COL2, ACAN) of joint sections at 8 weeks. Scale bar, 100 μm. **d** Quantification of COL2 and ACAN in cartilage tissues at 8 weeks (sample = 6 for OA+PBS group, sample = 8 for OA+tc-Mito group and OA+mc-Mito group). All data are presented as mean ± SEM. *p < 0.05, **p < 0.01, ***p < 0.001. P values were determined using one-way ANOVA (**b**,**d**).


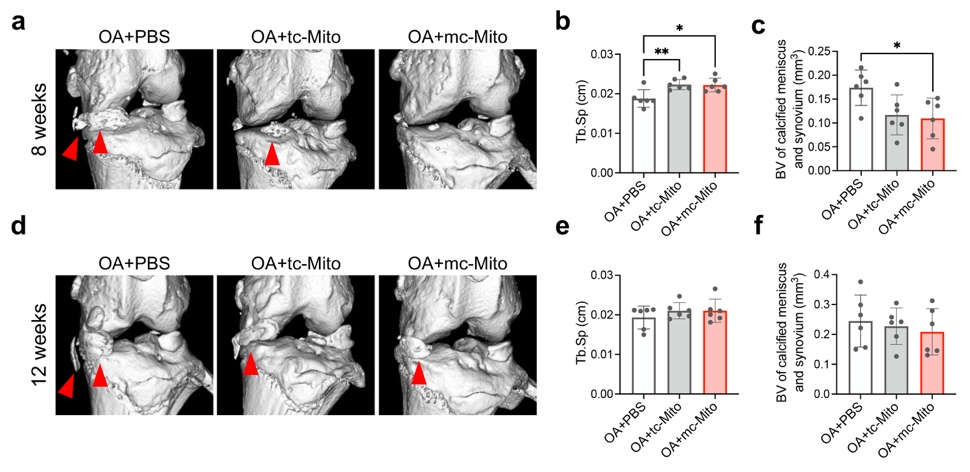


**Fig. S13** Mc-mitochondria treatment alleviates subchondral bone sclerosis and decreases periarticular osteophyte formation. **a** Three-dimensional micro-CT images of the knee joints at 8 weeks. Red arrows: osteophytes. **b** Quantification of trabecular separation (Tb.Sp) in subchondral bone at 8 weeks (sample = 6 for each group). **c** Quantification of the bone volume (BV) of calcified meniscus and synovium of the knee joints at 8 weeks (sample = 6 for each group). **d** Three-dimensional micro-CT images of the knee joints at 12 weeks. Red arrows: osteophytes. **e** Quantification of Tb.Sp in subchondral bone at 12 weeks (sample = 6 for each group). **f** Quantification of the BV of calcified meniscus and synovium of the knee joints at 12 weeks (sample = 6 for each group). All data are presented as mean ± SEM. *p < 0.05, **p < 0.01. P values were determined using one-way ANOVA (**b**,**c**).

Table 1. Human samples information.

| Individual | Age (years) | Gender | Disease | Sample location | Figures |
| --- | --- | --- | --- | --- | --- |
| Human adipose-derived stem cells | 56 | M | Fracture | Thigh fat | Fig. 2a(#1), 2b, 2c and 2k |
|  | 64 | M | Osteoarthritis (joint replacement) | Thigh fat | Fig. 2a(#2), 2b, 2c and 2k |
|  | 72 | F | Osteoarthritis (joint replacement) | Thigh fat | Fig. 2a(#3), 2b, 2c and 2k |
|  | 26 | M | Fracture | Thigh fat | Fig. 1c, 1d, S1, and S3 |
|  | 10 | M | Fracture | Thigh fat | Fig. 1e-j, 2d-j, 3, 4, 5, S2 and S4-9 |
| Human osteoarthritis chondrocytes | 64 | F | Osteoarthritis (joint replacement) | Osteoarthritis cartilage | Fig. S10 |

Table 2. Antibody List.

| **Primary antibodies** | | |
| --- | --- | --- |
| **Name** | **Source** | **Application** |
| CD34-APC | Biolegend, 343607 | 1:20 (FC) |
| CD45-PE | Biolegend, 368510 | 1:20 (FC) |
| CD73-PE | Biolegend, 344003 | 1:20 (FC) |
| CD90-APC | Biolegend, 328114 | 1:20 (FC) |
| CD105-PE | eBioscience, 12-1057-42 | 1:20 (FC) |
| Mouse anti-TOMM20 | Abcam, ab283317 | 1:500 (IF) |
| Rabbit anti-Ki67 | Abcam, ab16667 | 1:250 (IF) |
| Rabbit anti-GM130 | Proteintech, 11308-1-AP | 1:200 (IF) |
| Rabbit anti-Calnexin | Proteintech, 10427-2-AP | 1:200 (IF) |
| Rabbit anti-AMPK | Abcam, ab32047 | 1:3000 (WB) |
| Rabbit anti-phospho-AMPK | Cell Signaling Technology, 2535 | 1:1000 (WB) |
| Rabbit anti-ATP5A1 | Proteintech, 14676-1-AP | 1:2000 (WB) |
| Rabbit anti-UQCRC1 | Proteintech, 21705-1-AP | 1:2000 (WB) |
| Rabbit anti-SDHB | Proteintech, 10620-1-AP | 1:2000 (WB) |
| Rabbit anti-MTCO2 | Proteintech, 55070-1-AP | 1:2000 (WB) |
| Rabbit anti-TFAM | Proteintech, 22586-1-AP | 1:5000 (WB) |
| Rabbit anti-TOMM20 | Proteintech, 11802-1-AP | 1:5000 (WB) |
| Rabbit anti-LC3 | Proteintech, 81004-1-RR | 1:2000 (WB) |
| Mouse anti-β-Actin | Proteintech, 66009-1-Ig | 1:5000 (WB) |
| Mouse anti-GAPDH | Proteintech, 60004-1-Ig | 1:5000 (WB) |
| Mouse anti-COL2 | Santa cruz, sc-52658 | 1:50 (IHC) |
| Rabbit anti-ACAN | Abcam, ab36861 | 1:200 (IHC) |
| Acti-stain™ 488 phalloidin | Cytoskeleton, PHDG-1 | 1:500 (IF) |
| **Secondary antibodies** | | |
| **Name** | **Source** | **Application** |
| Goat anti-Rabbit 488 | Invitrogen, A11008 | 1:500 (IF) |
| Donkey anti-Mouse 488 | Invitrogen, A21202 | 1:500 (IF) |
| Goat anti-Rabbit HRP | Jackson, 111-035-003 | 1:500 (IHC);  1:3000 (WB) |
| Goat anti-Mouse HRP | Jackson, 115-035-003 | 1:500 (IHC);  1:3000 (WB) |

FC, flow cytometry; IF, immunofluorescence; IHC, immunohistochemistry; WB, western blot

Table 3. Primer sequences for RT-qPCR

| Primer Name | Base sequence |
| --- | --- |
| Human-ATP5A1-F | GTATTGCCCGCGTACATGG |
| Human-ATP5A1-R | AGGACATACCCTTTAAGCCTGA |
| Human-UQCRC1-F | GGGGCACAAGTGCTATTGC |
| Human-UQCRC1-R | GTTGTCCAGCAGGCTAACC |
| Human-SDHB-F | ACAGCTCCCCGTATCAAGAAA |
| Human-SDHB-R | GCATGATCTTCGGAAGGTCAA |
| Human-GAPDH-F | TGACGCTGGGGCTGGCATTG |
| Human-GAPDH-R | GGCTGGTGGTCCAGGGGTCT |
